# Caring Under Fire Across Three Continents: The Hadfield-Spears Ambulance, 1941–1945

**DOI:** 10.1093/shm/hkad010

**Published:** 2023-04-09

**Authors:** Laure Humbert

**Affiliations:** Senior Lecturer, University of Manchester, Department of History, School of Arts, Languages and Cultures, Samuel Alexander Building, Oxford Road, Manchester M139PL, UK

**Keywords:** French resistance, attacks on health care, allied medicine, Second World War

## Abstract

During the Second World War, the Hadfield Spears ambulance took care of around 22,000 wounded and/or sick patients across three continents. This article analyses how military attacks and instances of violence impacted on the psychological, emotional and physical health of those attending the wounded within this mobile unit. While historiography of allied medicine develops apace, analysis of the Free French health service remains rare. Yet the history of the Hadfield Spears ambulance provides a fascinating window into the neglected issue of attacks on healthcare in wartime, as well as a fresh scope for combining macro and micro perspectives. The deployment of both approaches suggests potent ways to connect intimate responses to attacks to broader histories of allied frictions and cooperation. Crucially, it offers rich insights into the development of a transnational ‘ethos of stoicism’, which helped to sustain the hospital’s community, in a fraught allied diplomatic context.

In October 1944, a Commandant of the French forces declared that if his soldiers had to pay the ultimate price of war, their preference was to die in the warmth and care of the international Hadfield Spears Ambulance unit.^1^ From March 1941, when it embarked for foreign services at Greenock to June 1945, when it was disbanded after the victory parade in Paris, the Hadfield Spears Ambulance followed French resistance troops across Asia, Africa and Europe and cared for around 22,000 wounded and/or sick patients.^2^ Throughout these four years, this medical unit crossed 21 territorial or maritime borders and operated in 36 different sites. Directed by an American philanthropist (Mary Borden, also known as Spears) married to a British Army Officer (Edward Louis Spears), it was made up of surgeons and doctors belonging to the French resistance, volunteers in the British Friends’ Ambulance Unit (FAU), ambulance drivers from the Mechanised Transport Corps (MTC), British trained nurses and orderlies from Cameroon and what was then French Equatorial Africa. A veritable ‘tower of babel’, in addition to multiple languages and nationalities, the unit encompassed different practices of war medicine, and distinct surgical and nursing traditions. Despite displaying Red Cross symbols, the Ambulance facilities were repeatedly bombed, shelled and machine-gunned. Its staff shared some of the danger and suffering of front-line combatants, withstanding the heat of the Western Desert (1942) and the freezing cold of the Vosges Mountain in France (1945). Although they performed a crucial service to de Gaulle’s Free French Forces, the Hadfield Spears did not receive official recognition for its contribution in the form of a collective medal after the war, unlike other medical units.

Almost from its inception, Free French authorities struggled to accept the ambulance’s *directrice* and the symbolism that her name provided.^3^ Mary Spears was not only *a woman* and a *foreigner:* she was also the wife of the ‘traitor’ Edward Spears, suspected of carrying hostile policies as head of the British official mission to the Free French. At the start, Edward Spears seemed a close Ally to the Free French.^4^ In June 1940, shortly after the defeat, he lobbied the British government on behalf of de Gaulle to obtain a formal recognition of the Free French Forces. Yet, he rapidly became ‘the enemy of the man he saved’, in Mary Spears’ own words.^5^ His relation to de Gaulle began to unravel after the failed Dakar expedition in September 1940 and fully broke off in the summer of 1941 after de Gaulle overturned the Acre Armistice in Syria.^6^ The tense relationships between de Gaulle and Spears had a significant impact on Mary Spears, who wondered whether she should continue to support the Ambulance, and on the day-to-day working of the unit.^7^ Although Spears’ influence over the running of the Ambulance gradually decreased in terms of funding and decision-making, French authorities threatened to dismantle the unit in 1944.^8^ And yet, this article posits that a distinctive international community emerged in the Ambulance on the ground, in which gender, professional and national boundaries were stretched under the pressure of enemy fire to create new ties of affect that outlasted the war.

In the increasingly professionalised world of Allied medicine, the Hadfield Spears Ambulance was an oddity, sitting between the ‘military’ and the ‘voluntary’. Its status was regulated by a note signed by de Gaulle, the head of the Free French Forces, and Mary Spears, on 22 February 1941.^9^ This note stipulated that this Ambulance was an ‘autonomous formation’, able to be deployed at short distance from the firing line and provide essential assistance in surgical triage for Free French units (in cooperation with the Free French Surgical Ambulance). Within the Hadfield Spears, a French chief medical officer was responsible for the military personnel, which was paid for by Free French forces.^10^ Mary Spears oversaw British and American nurses, paid the nurses and supervised the *Friends Ambulance Unit* volunteers, allowing for a ‘greater degree of latitude and self-government’ than in purely military establishment according to the FAU volunteer Michael Rowntree.^11^ In January 1942, the British represented nearly half of the personnel of the Ambulance.^12^ In this international unit, French medical officers had to negotiate the bleak reality of French Resistance’s dependence on its Allies. De Gaulle’s movement was entirely dependent on the British for material, logistics and financing. The story of the Hadfield Spears reflects the broader difficulties of the integration of Free French units in a British-led coalition, before their integration into the recomposed French Army after 1943.^13^ These units had to overcome differences of language, culture, equipment, and training. In spite of significant tensions, the Ambulance staff augmented throughout the war, and its role evolved from being a relatively small surgical mobile unit (with 47 staff and a capacity of 80 beds) to a much larger mobile ‘medico-surgical’ one, staffed with 208 personnel and composed of a *maison mère* (with a capacity of around 300 beds) and several forward surgical units (*poste avancé*), at the forefront of the evacuation chain.^14^

The variety of the spaces in which the Hadfield Spears operated, and the multiplicity of attacks that it faced, profoundly shaped the Ambulance’s cultures of caregiving and personal relationships between carers and combatants. The memoirs and oral testimonies of Hadfield Spears staff contain rich descriptions of the intimate attachments of friendship between carers, from various national, professional, religious and social backgrounds, as well as between carers and combatants. At first sight, it is tempting to explain what made medical workers ‘hold on’ under attacks by referring to basic primary ‘group theory’. According to this theory, combatants fight for their immediate group rather than abstract ideals and high principles (such as that of ‘nation’ or the liberation of France).^15^ Yet, coping mechanisms amongst this medical community were also markedly different from that of regular military troops. Under fire, as a French resister and medical officer noted in his memoirs, doctors, mechanics or stretcher-bearers, were neither ‘helped, like combatants, by the ‘fury of the combat’ nor by ‘the violence of the action’.^16^ Courage, for this French surgeon, was both the fear of ‘not being there in the next moment’ and the ‘ability, despite of this fear, to do what had to be done and remained calm in front of wounded who needed comfort’.^17^ This observation challenges us to consider further how trained and untrained medical workers responded to instances of violence, negotiated contradictory obligations of care, and faced ethical dilemmas.

To answer this question, this article traces the many strands of what I am calling a transnational ‘ethos of stoicism’, which emerged in this hospital as it followed French resistance troops through the Middle East, Africa and Europe.^18^ I understand ‘ethos of stoicism’ as a shared moral expectation emphasising self-control, abnegation and psychological detachment when looking after the wounded and sick in the face of events that could trigger fear, isolation and physical and mental trauma. This ethos manifested itself in myriad ways, through acts of bravery but also in the most intimate of care relations.^19^ Like other moral stances, it did not ‘transcend the world of everyday relations and experiences’, but rather ‘emerge trough and as part of them’.^20^ Under the laws of war, the c guiding ethos of the unit was to treat the wounded alike irrespective of their nationality. However, as this article explores, this ideal was often difficult to maintain on the ground. The ‘ethos of stoicism’ did not erase the tensions and contradictory bonds within and between the various ‘emotional communities’ of the Ambulance. This ethos of stoicism cut across what Barbara Rosenwein has called in a different context the various ‘emotional communities’ of the Ambulance, constituted by the Free French surgeons, the FAU, MTC drivers, nurses or colonial orderlies.^21^ Each of these ‘communities’ built on distinct (if not radically different) shared norms and expectations of feelings. For instance, the Free French were committed ‘body, mind and soul to their fighting’, while the FAU were Christian and pacifist, belonging to a long Quaker tradition, which combined witness against war and service to those in need.^22^ This transnational ethos of stoicism was neither a static system of shared values, nor the only commitment by which medical workers lived.^23^ Crucially, it was not inherently positive: it could be coercive as well as supportive. To put it another way, it had its ‘dark sides’: the ethos of stoicism stressed toughness and the ability to remain in control at all times, making it difficult to openly voice distress, grief and discomfort.^24^ Yet, this shared ethos was central to forming a successful (though not inclusive) transnational community, to individual’s coping mechanisms and to the ways in which they later remembered and narrated their wartime experiences.

Despite the rich historiography on Allied wartime medical and nursing services, the history of the Free French health service has received sparse treatment.^25^ This history, however, is worthy of particular attention because it shed new lights on what Mark Harrison has called the ‘victory of Allied medicine’ and provides a scope for combining macro and micro perspectives. Harrison attributes this victory to the relationships between medical and combatant officers, the superiority of Allied sanitary arrangements (based on British army’s greater experience in fighting in hot climates) and the cultivation of active citizenship in matters of health.^26^ This cooperation between medical and combatant officers was less smooth in the French resistance, particularly in the Western Desert. In the word of Vialard Goudou, chief medical officer of the First Free French Brigade in 1942, in the French headquarters ‘they understood nothing about medicine, less than nothing about surgery’.^27^ Facing acute material shortages, Free French medical officers drew on the experiences of the Rif War and the Spanish Civil War to organise the evacuation of their wounded.^28^Non-conformists and away from the direct supervision of the Londonian Free French Health Direction, they enjoyed a great degree of latitude and transformed Free French medical units into spaces of knowledge exchanges and experiments.

For historians of the French resistance, the history of HS offers a windows into the ways in which gendered discourses informed cultures of medicine and nursing within the movement.^29^ Guillaume Piketty has employed emotion as an analytical tool to better interpret and understand the motivations and daily life of French resisters.^30^ According to him, there might have been more than one way to *feel* a Free French, but the notions of elite combat and man-to-man bonding were at the core of Free French manliness, even for those who had rallied to the Free French cause in Africa or those, like doctors and surgeons, who did not bear arms.^31^ Within the French Resistance, the line between medic and soldier was blurred. Women were kept peripheral to the ‘combat-masculinity’ core of the Free French. Significantly, four surgeons were awarded the title of *Compagnon de la Liberation* for their courage and *sang-froid* during battles, the highest and most prestigious decoration for members of the resistance.^32^ This Free French dependency on a masculine image and man-to-man bonding made it difficult to accept the authority of a ‘Lady’.^33^ While welcoming the presence of nurses, Free French medical officers disparaged the ‘amateurish’ approach of *Lady* Spears.^34^ Vialard Goudou sarcastically mocked Henri Fruchaud (the surgeon chief of the Ambulance in 1942) writing in his diary ‘It is not worth studying medicine for seven years… if when war comes, one needs to remain under the tutelage of generals’ wives…’.^35^

While the history of the French resistance has for a long time been written essentially as a ‘French story’, this article demonstrates that the Hadfield Spears tied bonds between persons with different sexes, national identities, and approaches to the war. British FAU orderlies were conscientious objectors (COs) who refused to bear arms on political, moral or religious grounds. FAU rejected British cultural idealised wartime notions of the ‘military hero’.^36^ Their moral stand made it difficult to cooperate with the armed forces and to accept the compromises of battles. Yet, as Tobias Kelly observes, despite their pacifist conviction and commitment to medical impartiality, the ‘battlefield still represented the ultimate measure of bravery’.^37^ For the very few British and French female nurses and ambulance drivers involved with the hospital, known within the hospital and the Free French movement as the *Spearettes,* the stake was to convince the British and Free French conservative male military elites that women had their place near the front line.^38^ Showing the same resilience, courage, calm and flexibility as male combatants was essential to breaking down prejudices against women working at the front and gain their place in the conservative landscape of Allied military medicine.^39^

## Silences in Narratives of Medical Personnel

In order to grasp the emotional complexities of the experiences of medical staff under fire in a transnational perspective, I turned to a heterogeneous corpus of sources, which includes archival records, private diaries and correspondence, published memoirs and oral interviews conducted by Lyn Smith between 1987 and 1993 for the Imperial War Museum.^40^ Contrary to other medical spaces of the French Resistance which have left sparse archives, a rich paper trail exists about the Hadfield Spears and its different communities.^41^ However, this material needs using with care. Historians have warned about the risks of using oral histories recorded in the 1990s to examine every day social practices in the 1940s, as the interviewees were ‘reconstructing their youthful selves back’.^42^ Interviews are always dialogic and intersubjective: there are shaped by the interactions between the interviewee and the interviewer.^43^While acknowledging the need to handle these different types of personal narratives carefully, this article takes the line that both the fragility and creativity of memory is itself a resource. Read closely, these sources provide a fascinating window into how these men and women, who were not combatants but often identified with them, ‘composed’ their masculine and feminine selves.^44^

As many historians have noted, diaries, oral testimonies and memoirs do not give transparent access to the sentiments and emotions of those who wrote them.^45^ First, life-writings are shaped by the prevailing cultural discourses and codes of the time. Memoirs have been central to the history of the French resistance. Since the end of the conflict, former French Resisters wanted to tell their stories and provide their own account of the history of the Resistance. They rewrote themselves, conferring new meanings to their wartime experiences and creating important myths and legends, some of which continue to prevail in the historiography. Their accounts often emphasised intense experiences of solidarity and fraternity, as well as a profound and deep commitment to disobey. As Guillaume Piketty observes, reading emotion in resisters’ personal writings requires thinking about self-censorship and taboos.^46^ This is particularly noteworthy for caregivers of the French resistance. In a historiography and collective memory dominated the voices of former resisters, medical officers have tended to be remarkedly silent about the wounds of war.^47^ This is all the more significant as the ‘Resistance’ has a particular place in the French public imagination and often stands as an example of absolute virtue.^48^

Silences in medical personnel’s accounts also ought to be scrutinised in the context of mid-twentieth century medical and nursing education. As Jane Potter and Carol Acton demonstrate, a culture of silent stoicism shaped medical educational traditions since the late 19^th^ century. This meant that care-givers tended to internalise a hierarchy of suffering and represent their own emotional pain obliquely, foregrounding in their writings the story of the wounded for whom they cared.^49^For instance, In Bir Hakeim (Libya), in the heat of the Western Desert and the midst of what would become a legendary battle for the French resistance, frontline surgeons faced an acute dilemma over evacuating the wounded. On 9 June 1942, 5 bombs fell on the surgical post, killing 15 patients and 5 nurses, and, during the night of 10 and 11 June, surgeons had to abandon the most wounded during the final evacuation. The French surgeon Jean Vernier later recalled ‘heroic night, nightmare for all, when one had to leave lying there, to the care of the Germans, the wounded deemed not transportable, horrendous night, that the witnesses, surprised to have survived, did not like to talk about’.^50^

By reading between the lines, being attentive to silences and looking for not-fully conscious affective states, I attempt to go beyond prescriptions and standards of acceptable emotive responses to find evidence of messy and discordant emotions. Letters, diaries or memoirs are emotional spaces in their own right, sites where individuals attempted ‘to work out and articulate [their] own hybrid identity’ as medic or nurse.^51^ In other words, historians must not only examine the content but also the materiality of these texts, considering the ‘use of space, erasures, stumbles and handwriting’ that hint at their writer’s emotional state.^52^ This approach is particularly fruitful for the analysis of Mary Spears’ diary, which differs markedly from her published memoirs *Journey Down a Blind Alley* (1946).^53^ In her First World War writings, Mary Borden/Spears used modernist techniques, including intense imagery, ruptured narrative and obsessive repetitions, to contest the official and militarized views of the war.^54^ By contrast, *Journey Down a Blind Alley*, is a lot more conventional in style and content. But Spears’ diary reveals a great deal about her nagging doubts over what was the best course of action as the conflict between de Gaulle and her husband worsened. She often wondered whether she should follow ‘her’ ambulance or stay put in Beirut with her husband to counter the affection of his mistress. In June 1945, she reflected very critically on her contribution to the war, confiding ‘the fine record of the Unit is in no sense due to me – and I will never stop wondering at how little I have done for it with what good results’.^55^ This is in sharp contrast with the heroic narrative that appears in *Journey Down a Blind Alley*.

It remains to be said that some female voices are missing, such as that of the only Free French female doctor. In the highly hierarchical world of war medicine, surgery was regarded as a masculine job, requiring male intelligence and strength.^56^ In 1941–1942, the only female doctor was Louise Marie Lemanissier, an anaesthetist. Anaesthetist was considered an auxiliary and low-grade task.^57^ Lemanissier had to fight for the right to practice her profession, while insisting that her role remained auxiliary to that of men.^58^ There are more systematic gaps in the archival records too, particularly in relation to colonial orderlies. While we have many snapshots of what attacks looked, felt and smelt like for different European members of the Hadfield Spears, we can only see glimpses of the ways colonial orderlies survived them.^59^ In the archives, the perspectives of colonial orderlies are always mediated, their views appearing through the French or British colonial gaze. As Marisa Fuentes notes in another context, the persistent effects of white colonial power continue to constrain and control what can be known about them in the archive.^60^ In the hospital, colonial orderlies were confronted with systemic racism, facing both codified structures of racial exclusion and *de facto* practices of discrimination. What follows, then, is necessarily an incomplete tale of the ways in which responses to violence were culturally prescribed, influenced by age, gender, ethnicity, in part because of the disparate nature of the sources.

## Facing Fire

Since the 1864 Geneva convention for the Amelioration of the Condition of the Wounded (and the subsequent conventions of 1906 and 1929), medical personnel and patients were protected by international treaties. Yet, in first-hand accounts, military attacks were often described as part of the normal life of the hospital. Neither international legal ideas about medical neutrality nor the Geneva Conventions were used when describing the violence that the medical personnel faced.^61^ In both the hospital’s official logbook and personal narratives, it is difficult to distinguish between attacks (bombings, explosions, shootings, gunfire, assaults on personnel, theft of medical supplies, etc.) deliberately targeting or unintentionally impacting the hospital. Aerial bombing was particularly intense in June 1941 in Syria, in the spring of 1942 in Libya, in the autumn of 1942 near El Alamein, in April-June 1944 in Italy and in the summer of 1944 during the landing in France.^62^ Even high-profile air attacks are rarely commented on, except in February 1942, when the leader of the FAU died or in May 1944, in San Giorgio, when three members of the hospital were severely wounded following a raid.^63^

For the *Directrice*, Mary Spears, and the French professor of surgery Henri Fruchaud who was influential in shaping its early development, the medical and surgical successes of the unit depended on its ability to follow closely the battle, even if it jeopardised the protected status of the hospital. On several occasions, FAU volunteers complained about the misuse of ambulances and protective emblems and the placement of the hospital near the ammunition, particularly in the Western Desert (1942) or in Italy (1944).^64^ The FAU volunteers were the only ones to regularly evoke the Red Cross Convention of 1929 and to wear their Geneva Identity card and a stamped Red Cross Brassard. They did not accept unreservedly the Geneva Convention, for they disagreed with the use of weapons to protect patients, staff or medical equipment.^65^ In 1942, they refused to transport arms in their vehicles. This brought about ‘one of the few major disputes’ between the FAU volunteers and Free French surgeons.^66^ Fruchaud regarded their refusal ‘as unsatisfactory evidence of our civilian status and freedom from army discipline’ in the words of FAU volunteer.^67^

Both Spears and Fruchaud had been profoundly impacted by their medical experiences during the First World War and the memories of wounded men being evacuated too late. Mary Spears had won British medals and the French *Croix de Guerre* for her nursing work, while Fruchaud had followed closely the development of war surgery since the First World War and notably during the Spanish Civil War. He was familiar with the work of Josep Trueta who revolutionized the treatment of fractures. For Fruchaud, war surgeons had to transform war wounds most rapidly into surgical wounds with the French method of *épluchage* (the excision of a wound in its entire extent) and the subsequent application of plaster to immobilise the wound.^68^ According to Fruchaud, the surgeon had to intervene within forty-eight hours (and ideally sooner) of the infliction of a war wound to the muscles and bones (more particularly with those of the limbs, buttocks and shoulder). ‘Early operation, which we consider vital, cannot be assured unless the surgeon and his apparatus can follow the battle’.^69^ Providing early treatment near the front line was not only vital for surgical and medical reasons: it also offered crucial support for the morale of the combatants.^70^

For Fruchaud, the ability to operate in optimal conditions prevailed over the necessity to mitigate the danger of aerial attacks. In the autumn of 1941, he designed an operating theatre made of two air and sand-tight adjacent lorries, a surgical and a pharmaceutical one.^71^ This operating theatre could neither be buried nor camouflaged, thus representing an easy target for the enemy. For the French surgeon Vialard Goudou, this operating theatre (called the ‘cathedrale’ by the Free French) was an *absolute* tactical blunder.^72^ According to Vialard Goudou, Spears and Fruchaud defended an organisation that was ‘ill adapted to the needs of the Free French group and to the operational realities of the desert war’.^73^ In his diary, Vialard Goudou frequently mocked ‘Fruchaud theories’, deriding his high ambition and egocentric personality. Despite important frictions between Free French medical officers, they agreed with British authorities that women should be protected from the firing-line in the Western Desert.^74^On 29 January 1942, as the hospital moved forward, Fruchaud and Spears received the order to send all female nurses back to Tobruk in the 62th General British Hospital and to detach a small surgical unit, headed by Fruchaud, in Bir Hakeim.^75^Free French elites saw the incorporation of women in the British Army as both pioneering and threatening.^76^ The few women in military uniform in the Ambulance were expected to show the same resilience, courage, calm and flexibility as the men, but neither to challenge the hierarchies of wartime surgery nor threaten the virility of their male colleagues.^77^ As Cynthia Enloe argues, the position on frontline nursing was full of contradictions and ambiguities.^78^ For one thing, women’s presence in the desert undermined the military’s legitimising image of manhood. As Frances Houghton notes, the Western desert, ‘in its very hostility towards mankind’, was interpreted as uniquely stamping soldiers with prized toughness and manliness.^79^ For another, military authorities were aware of the gendered position of women as ‘morale booster’ to bolster men’s resolve to fight. Female nurses themselves did not see their position as women as devaluation of their worth but rather as part of their importance in a war zone.^80^

In Bir Hakeim, a highly significant position for the Free French, minefields surrounded the forward unit, installed in the Western Desert.^81^The hospital received the order to bury everything and to prepare for powerful aerial attacks.^82^ According to the nurse Nancy Smith, colonial orderlies did the heaviest digging.^83^ The FAU volunteer Nev Coates wrote in his diary that the French were ‘really nuts’, mixing up the hospital with artillery and ammunition dumps.^84^ Each staff had an individual hole (bivvie) in which to hide in case of air attacks, but the main operating theatre, the ’cathedrale’, could not be buried underground. During the main battle of Bir Hakeim (26 May -11 June), the forward unit directed by Pol Thibaux undertook an ‘excursion to hell’. In his diary, the doctor Paul Guénon wrote ‘Bullets, Shells, Bombs… there are moments when we envied the dead who were resting’.^85^ From 2 June, it became impossible to evacuate the wounded at the rear. The surgeons operated all the wounded: at equal degree of emergency, British patients had the priority, followed by the Free French and then Germans.^86^ The operating theatre was destroyed by German aviation on 10 June 1942.^87^Fifteen severely wounded patients and five nurses were killed.^88^ After the destruction of the main operating theatre and the killing of all patients, Guénon had to operate on the wounded in his own hole, where there was only space for him and the wounded. During the final evacuation, he was forced to abandon his wounded patients, in order to save his own life and that of other (but less) injured comrades. Guénon notes: ‘Horrendous sights: the wounded that we have to abandon. Oh! These screams… taking care of them, means to commit suicide’.^89^All medical staff survived the last phase of the battle, and only one doctor was wounded.^90^ Guénon’s diary hints at his exhaustion, due to sleep deprivation, the paucity of water and the encirclement of French troops by the enemy.^91^ But there is no mention of psychological collapse. Guenon’s construction of his work and stoic endurance bears comparison with the narratives of combatants, with particular emphasis on courage, morale and brotherhood with those patients that could be saved.

In Tobruk, the General Hospital was also heavily bombed in the spring of 1942. Like their male counterparts in Bir Hakeim, women were required to face air war stoically.^92^Nursing sisters, trained in the British hospital system, and ambulance drivers were well versed in the need to remain externally calm in any circumstances.^93^ Nurses gave meaning to their experiences of air attacks by referring to common cultural tropes and First World War memories. The Ambulance’s staff labelled the bombing of Tobruk the ‘African Verdun’, consciously drawing parallels with the experiences of First World War veterans.^94^ They were influenced by popular representations of their predecessors, which casted nurses as icons of patriotic femininity embodying the feminised version of male military sacrifice.^95^ Heavy bombing rarely prompted outrage, as air strikes on hospitals were expected to happen, even if it was in violation of the Geneva conventions. In 1943, the ICRC representative Jean Pictet noted that the ICRC received many complaints about the bombing of hospitals and torpedoing of hospital ships, but that there was little that the organisation could do in practice.^96^ Further, aerial bombing technologies lacked precision, and there was a considerable margin for error when aiming at a target.^97^

Despite their proximity to the frontline and defiance of female stereotype of vulnerability, the *Spearettes* did not fundamentally question French and British military commands’ gender ideology. They had been very carefully selected by Mary Spears for their professional skills, efficiency and moral grounds, and were held in very high esteem by the Free French medical officers for their courage.^98^ Class and Britishness were very important here: the upper-class origin of ambulance drivers protected them from accusation of dubious morality and sexual immorality associated with lower class militarised women, such as the ATS and contributed to their glamour.^99^ For Vialard-Goudou, they were capable of rolling their sleeves up and getting their hands dirty, unlike French women.^100^ In contrast to allegations made about other women’s service, no explicit associations were made between the *Spearettes* and sexually deviant behaviour: neither nurses nor ambulance drivers, though wearing mannish and military clothing and carrying work requiring physical strength, stretched the moral boundaries of proper womanhood.^101^

As the war progressed, French surgeons came to acknowledge how crucial women nurses and ambulance drivers were to the Ambulance’s operation. In 1944, during the Italian campaign, the Ambulance was confronted again with serious mortar, cannon and shell attacks, the tents were riddled with shrapnel and the terrible smell of decomposing bodies.^102^Male stretcher-bearers and female ambulance drivers took on a particularly dangerous and exhausting job on par with that of front-line combatants. The attacks were so violent that French soldiers wanted to launch another attack to avenge the attacks on the Ambulance.^103^ The FAU Eric Harper recounted that the hospital received around 20 shells in one afternoon, noting in his diary that ‘the wounded, their nerves sadly affected, [didn’t] take kindly to the shelling which is not surprising’.^104^ According to the FAU David Rowlands, ‘it was the only time […] that the hospital was picked up as a target’.^105^ The FAU Roy Ridgway and Eric Harper both admitted in their diaries ‘feeling panicky’ and ‘being in a very nervous state’.^106^ The nurse Evelyn Fuhlroth remembered that she thought she would not survive the attack. She then explained that she received a *Croix de Guerre* [French military medal] following the battle, a decoration that she was very proud of. Fortunately, there were no serious casualties. It was, in Rowlands’ words, ‘an extraordinary piece of luck’.^107^ Only three members of staff were wounded.^108^ Ridgway commented on his externally calm colleague, the surgeon Jiberry ‘He was putting a needle – he was about to give a blood transfusion – putting a needle into a vein and a big chunk of shrapnel felt at my feet and he did not flinch and he went on doing this very steady and I had terrific admiration because a few more inches it could have killed him or it could have killed me’.^109^ In his official report, the chief medical officer Vernier concluded that the delivery of care was not affected by these air attacks. The staff treated 1964 wounded and ill patients during the campaign for the majority French, but also 75 Italian civilians and 40 German POWs.^110^ The female staff of the hospital gained formal recognition of their courage in the forms of military medals and deployment in dangerous situations, of which they were really proud.^111^ Significantly, in the summer of 1944, after the Italian campaign, two women (Miss Pryke et Miss Howell Evans) were part of the “elite” commando unit (formed of seven members) that landed in Provence in August 1944. This was a highly symbolic decision, attesting to the value attributed to his feminine staff by the surgeon Vernier. His report was particularly laudatory about the courage of these two women under fire and in condition of total insecurity.^112^ ‘They held on under the bombs, in the same manner that Franka Kohen, a Polish Jewish nurse, in her operating tent and under direct artillery bombardment did in San Giorgio de Liri’.^113^

In short, due to its proximity to the frontline, Hadfield Spears staff expected the unit to be attacked and accepted the subsequent risks, or at least this is what they recounted in their post-war testimonies.^114^On the whole, the number of staff casualties directly resulting from direct military attacks remained limited. In total, according to French official estimates, 55 members of the Health Service of the First French Division died or went missing.^115^ Within the Hadfield Spears, seven male members of the hospital were killed (while in service) and over 40 were severely injured or fell ill.^116^ If we compare these figures to the risks faced by other members of the French resistance, whether combatants or members of *bataillon médicaux,* the death rate was considerably lower.^117^ While they were not confronted by constant danger, they nevertheless frequently witnessed death and pain. As a FAU member notes, ‘The worst part of an attack for those at the rear is waiting and wondering how many of their friends will be brought into the hospital, perhaps to pant away their lives in a hot tent ward’.^118^ Like many other medical workers, witnessing the pain of others, they were often unwilling to claim any pain and suffering for themselves.^119^

## The Ethos of Stoicism

The ethos of stoicism manifested itself in myriad ways, through bravery acts but also in the most intimate of relations, when holding the hand of a dying comrade, writing his last letter, or making a hasty cross to bury him. In February 1942 in El Azragh, Nev Coates helped a dying patient to write his last letter to his mother. ‘It was a moving job to take down his letter, saying that he wasn’t bad and that he was thinking of her. Difficult, too, to stay in the tent and appear to be cool and pacify the wounded when the German planes came low, with the panicking dying man trying to get up off his stretcher’.^120^ Cultivating detachment and a certain distance with patients was an essential part of medical and nursing training.^121^ Since the late nineteenth century, medical and nursing publications had promoted cold compassion and the unsentimental delivery of care.^122^ Excessive emotions were perceived as a threat to rational decision-making. Medical workers (from surgeons to voluntary first aid nurses) certainly internalised in some ways this image of the dispassionate and professional medical worker. But as Nev Coates’ testimony suggests, the experience of fire reveals complex emotions underneath the externally calm appearance.

In her First World War writings, Mary Spears powerfully captured the dilemmas faced by frontline nurses, splitting the nurse into two figures, one driven by self-control and technical efficiency and another one by ‘emotional involvement and the threat of hysteria’.^123^ On the one hand, as Ana Carden-Coyne notes, First World War nurses were depicted as ‘brutal women’, yielding ruthless power over wounded bodies and inflicting pain.^124^ On the other hand, Christine Hallett has demonstrated that nurses helped wounded men healed, by ‘containing their trauma’ and ‘creating safe boundaries within which healing could occur’.^125^ Sarah Chaney has observed a change through the 1920s and 1930s, largely as a direct response of the war – towards more emphasis on fortitude in nursing training in Britain. The ideal nurse came to be associated not simply with an ability to hide one’s true feelings, but also with a capacity to put on a display of bravery.^126^ In the inter-war period, nurses were taught to exhibit a restrained ‘sympathy’ towards patients: in other words, they were expected to ‘enter within the patient’s experience of pain’, but not project their own feelings onto them.^127^ In theory, the delivery of care thus needed to be controlled, orderly and ‘unsentimental’ in the Hadfield Spears. In practice, though, nursing under fire could play host to a wide range of more complex feelings including fear, pity and despair.

Despite a shift in feeling rules and expectations in nursing in the interwar period, these tensions between emotional detachment and involvement, and between violence and care, remained at the heart of nursing in the Hadfield Spears. In her oral testimony, the nurse Josephine Pearce recalled how in Deraa she spent her nights, armed only with a hurricane lamp, sorting out the wounded that she thought could be operated on from those who could not. In an explicit cultural reference to Florence Nightingale (“the lady with the lamp”), she admitted ‘I am afraid, some did die. Had we had more people to help these might have been saved’.^128^ She reminisced about holding dying men’ hands and letting them think that she was their *Nanny.*^129^As Ana Carden-Coyne observes, ‘the sensory and emotional effects of working with the wounded were deeply personal’, men ‘inscribing something of their pain’ in the minds and memories of the nurses that tended them, no matter how detached they had been trained to be.^130^

The’ethos of stoicism’ under fire was thus rooted in the training and professional cultures in which Hadfield Spears staff had evolved in the inter-war years. For surgeons, as Michael Brown, Thomas Schlich and James Kennaway have demonstrated, a culture of dispassion and emotional detachment permeated broadly throughout European surgical spheres from the late 18th century.^131^ If fear was considered as normal reaction, combatant and surgeons alike were expected to control it, particularly in battle.^132^ It is thus not surprising that many testimonies insist on the self-control and calm with which French surgeons continued to operate under the bombs.^133^ In her memoirs, Mary Spears described Fruchaud as a ‘tiger’ (perhaps in a reference to Georges Clemenceau) who ‘could and did work at lightning speed for twenty-four hours on end’.^134^ Mary Spears recounted the heroic performance of the French surgeon Pol Thibaux, who carried out twenty-one big operations during the last day of the Bir Hakeim battle ‘with bombs falling all around him’.^135^

Within a broader comparative context, this emphasis on self-control and fortitude was not confined to the experiences of medical workers in the Hadfield Spears. During the Second World War, the increased mobility of units contributed to aggravate the stress and difficulties of medical work. American and Wehrmacht military medical services gave similar descriptions of self-control, heroic sacrifice and fortitude during the Normandy battles following D-Day in 1944.^136^ Showing courage and fortitude was also crucial for FAU volunteers, as their refusal to take arms could be perceived as feminization and an abandonment of their masculine self.^137^ As Tobias Kelly argues, the FAU volunteers were driven by a determination to show others that they were not coward and that they were willing to make sacrifices for others. For all the talk around the notion of ‘temperate hero’ in Britain, the battlefield remained the best test of manliness.^138^ The expectation of courage under fire thus cut across different professional and ‘voluntary’ groups and was not specific to the Hadfield Spears units. Medical staff were even more reluctant than their First World War ancestors to acknowledge the psychological consequences of working in a ‘world of hurt’.^139^As Ben Shephard observes, while there was a progressive recognition of the effects of battlefield stress on combatants within the military establishment—expectations of stoicism under fire remained unchanged for medical staff and non-combatant men during the Second World War.^140^ What was specific to the Ambulance, however, was the bond that tied its people together.

This transnational ethos of stoicism was never simply abstract ideals of *sang-froid*, emotional detachment, and self-control. It runs through medical workers’ relationships with their colleagues and patients, even for surgeons who were traditionally portrayed as insensitive to patients’ suffering. Before joining the Hadfield Spears, Fruchaud carried out surgical operations on the front line at 2000 m altitude in the Eritrean mountains, two days’ walking distance from the base unit.^141^ The surgical post received a violent mortar attack and one of his colleagues was wounded. Fruchaud did not leave a diary rich in emotional expression but he honoured the memory of his friends - the pharmacist Rabaté killed in 1941 and the lieutenant Colonel Amilacvari in 1942 - in the preface of his treaty on war surgery, published in 1943. He showed a powerful sense of emotional intimacy with combatants, noting that his memories of the battle with Amilacvari in front of Kheren in Eritrea were amongst the most moving of his life.^142^ Even FAU volunteers felt the brotherly love that stretched between combatants and carers. They did not want to carry arms, but they were still committed to their fellow comrades, often preferring to be in the Forward Unit than in the base hospital at the rear.^143^ In his oral interview, Roy Ridgway reflected on his work in the resuscitation tent during the Battle of Monte Cassino in May 1944, dealing with the worst cases when the guns ‘were firing on both sides’. He commented on the horror of war, but also its ‘terrible beauty’ and the bravery, courage and terrific bonds that battle created amongst men.^144^

The HS thus combined very different kinds of wartime sacrifices, accommodating the pacifist consciences of FAU volunteers with the vibrant patriotism of Free French surgeons. In his diary, for instance, the surgeon Paul Guénon expressed his ‘total contempt for death’.^145^ He related a conversation with a British officer, in which they compared a peaceful and domestic death to a violent one: ‘I oppose the glory of a bullet in the head to the progressive and banal decay of the ill. But Bell prefers an illness, a comfortable room, a beautiful nurse, herbal teas and parents anxiously bending over the white bed…’.^146^ To be sure, the valorisation of death and sacrifice was not specific to the emotional cultures of the resistance.^147^ But what distinguished Free French doctors from conscripted military doctors was that death was neither the result of a military order nor a passive act of submission to military discipline. Death represented a search for excelling oneself, which gave meaning to life.^148^

On 14 February 1942, when the leader of the FAU volunteers Raymond ‘Nik’ Anderson was killed in his slit trench by a German bomb in El Azrag (near Bir Hakeim), Mary Spears wrote in her diary:

There is something peculiarly poignant about it’s being Nick. He was so gentle.[…] So charming. And so determined to stay with the forward section. He was really pursuing the good life, a life of sacrifice. He had given two pints of blood yesterday to an English captain – said he was all right – but must have fainted for he found himself lying on the ground… I was often hard with him. He was always very considerate to me – almost as a son would be – thoughtful and unselfish.^149^

Although Mary Spears struggled to emotionally understand the pacifist stance of FAU volunteers, she was deeply pained by the death of Nik. The FAU volunteer Eric Harper notes in his diary that Mike Rowntree brought back with him the news of his death and admitted ‘we cannot yet quite take it all’.^150^That day, the FAU volunteer Dave had tears in his eyes and Nev could not quite cope with the sound and shaking of the ground. ‘Nik hasn’t just died, or been killed – but it seems that he has been snatched away’.^151^Bearing witness to such a violent death lived on vividly in the memory of FAU volunteers, who all mentioned Nik’s death in their post-war testimonies. Although pacifists, they were confronted with a defining feature of combat: an intimate encounter with a shattered corpse, an experience that usually set combatants apart from others. Mary Spears wrote to Nik’s mother shortly after his death that she wished it ‘had been someone else—older—someone whose life, like mine, has been lived. He was exquisitely thoughtful of me always, almost like a son. I shall miss him.’^152^ In the Ambulance, like in many other mobile hospitals, medical workers often took on the responsibility to write condolence letters, sending bereaved families a description of their loved one’s last moments.^153^ The also looked after the graves of lost comrades: a year after Nik’s death, the FAU volunteer came back to visit his grave in the desert and found it ‘untouched’, wild flowers having grown around.^154^

The ethos of stoicism did not only reverberate through acts of military-type bravery, but also in the ways in which medical workers were able to bear injuries and illnesses themselves. In Deraa, the FAU volunteer David Rowlands recalled that most of the staff were suffering from dysentery, while ‘the casualties were coming in faster than we could cope with’.^155^ He suffered from exhaustion and struggled to cope with the scale of the devastation. There were stretchers as far as he could see outside the reception tent ‘with people with legs shattered and facial and throat injuries’. Admitting that the experience of looking after patients ‘was just more than one could stand’ yet that ‘they kept going’, he offered a vivid description of the tensions and contradictions within which this ethos is enmeshed. He recounted that the two surgical units, one led by Fruchaud the other one by Durrach, worked day and night in blazing sun and tearing wind.^156^

[T]his was just more than one could stand and yet somehow we kept going it seemed to be days and nights… and I always remember we ran out of orange boxes for Jacopin who was one of the Breton fishermen who came over to make hasty crosses for burying the dead. We had just to smash out these orange boxes and in these improvised sort of graves we put crosses of a kind up and this work went on and I remember that captain Asquin, who was one of the medical officer, was absolutely exhausted with dysentery and could scarcely keep going.^157^

The doctor André-Francois Lemanissier also notes in his memoir, that after ten days of looking after patients, he suffered a terrible dysentery and had to stop working. He comments on the exhaustion of his wife, the only female doctor and anaesthetist, working twenty hours a day without sleep. ‘I don’t know how she has managed to cope’.^158^ Shortly afterwards she suffered from a viral hepatitis. The campaign in Syria was all the more difficult that Free French medical officers had to grapple with operating on the bodies of Frenchmen shot by other Frenchmen. In his diary, the French surgeon Vialard Goudou powerfully captured his intimate encounter with a twenty-year-old French enemy body, the body of a *gamin*, exhausted, bloodless and with a feat in his grave.^159^His diary hints at the mental struggles that he faced when taking care of this fellow enemy French patient. In her memoirs, Mary Spears recounted that she too found it difficult. I feel again’‘, she wrote, ‘not the physical suffering of the men’s mangled bodies – that I was used to, it was an old story – but the festering pain of their minds’.^160^

If this ethos of stoicism helped to sustain the community of the hospital, it was not without its frictions.^161^As they attempted to defend their female power within the male-dominated medical hierarchy, female nurses and socially elite ambulance drivers tried to distinguish themselves from each other and fight for professional recognition. British nurses considered themselves as better trained and skilled than both Free French nurses and British ‘mock’ nurses, the ambulance drivers who had little medical expertise.^162^Nurses came from families who were often considered middle class but who did not have the financial resources to support unmarried daughters. The nurse Evelyn Cottrell recalled how the drivers called them ‘Nanny’. If nurses were happy with being called *nanny* by dying male patients, they refused this term when it came from their female counterparts, who had no qualification except their family connections. For Cottrell, the drivers did not contribute much to the work of the hospital, being more interested in going ‘off to see their very superior boyfriend’.^163^ This depiction of ambulance drivers as unskilled and pleasure-seeking amateurs fitted with older stereotypes inherited from the early twentieth century.^164^ The nurses could also be critical of the FAU volunteers, treating them as ‘probationers’ and ‘emptiers of bed-pans and urinal bottles’, which lowered at times the FAU morale.^165^

The ethos of stoicism sustained the Ambulance community, which was essential for the emotional and physical survival of individual staff. Yet this community was also fragile, in part due to the marked differences and tensions between its various groups. Although the experiences of medical care under fire brought white European staff into close proximity with colonial orderlies, it neither abolished the violent structure of colonial domination nor the unequal power relationship between white lower rank officers and colonial orderlies. In the hospital, colonial orderlies faced systematic racism: official instructions, informed by racist presumptions, prevented them from sharing meals and socialising with white members. In July 1941, a confidential note signed by the *Chef du personnel indigene* and Fruchaud stipulated that ‘one should not forget that only the less interesting elements’ are sent to our hospital.^166^ They did not wear the same clothing as the white members of the hospital and, in theory, were not allowed to socialise with white members. A FAU volunteer commented that their winter clothing, which had been captured from the Vichy stores in Syria was ‘pretty poor stuff and made them look almost comical’.^167^ As a result, they met with derision from some other *Tirailleurs* [term used to designed French colonial troops] which led to some protests within the Ambulance. The FAU leader Michael Rowntree admitted in his oral interview that, although they got ‘reasonable treatment’, ‘the French would certainly always give priority to European soldiers if there were any case of priority being necessary’.^168^ This anecdotal evidence opens up important questions for further research.

Care-giving in this extreme environment and under fire supported narratives of comradeship and bonding between FAU members and colonial orderlies. The work on the *poste avancé* was often hard and sometimes dangerous, but it brought unit members into informal and intimate contact. According to the official FAU history, ‘differences in languages were forgotten and curious tongue was developed known as “Spears”’, a ‘macaronic riot of English, French and Arabic’.^169^ ‘I am going to my tent to fetch my mess-tin’ would become ‘I am going to my gitoun to cherche my gamelle’.^170^ Michael Rowntree argues that the FAU treated the non-white orderlies in a more equal manner than the French.^171^ These humanizing encounters did not mean that violence and institutionalised hierarchies of difference disappeared completely. In the spring of 1942, Nev Coates related ‘some Tirailleur trouble’ in the hospital, hinting at the violence people of colour were faced with.^172^

Despite the sentiment of intimate brotherhood within the hospital, non-white colonial orderlies remained subject to racial stereotyping and the imperial gaze. In the eyes of the British and the French, wounds and the experience of fire could confirm stereotypes about subaltern masculinities and justified unfair and harsh treatments. French elites believed that ‘battle stress’ manifested itself differently among them, especially for those who were Muslim due to the alleged negative influence of Islam and supposed importance of superstition in the psychological disorders of indigenous soldiers.^173^ It is very difficult, if not impossible, to know how colonial orderlies understood their emotional experiences when operating under fire. Testimonies from FAU members suggest that working under fire created opportunities for humanizing encounters. Despite strict discrimination policies to separate colonial orderlies from their European counterparts, strong bonds were formed between FAU and non-white orderlies, who did the most menial tasks in the hospital, including digging, cleaning, and serving food, and occasionally the most dangerous (carrying wounded patients under the bombs in Italy).^174^ These colonial encounters could both reinforce and undermine stereotypes about the Other. The FAU Nev Coates wrote in his diary that he had ‘always thought of Arabs as being stoical, bearing hardship and pain with a stiff upper lip’, yet he discovered that ‘some of our Arab patients were quite childish with their complaining and demands for attention.’ He asked himself ‘Was it that their pain levels were low or that they had to demonstrate their emotions which were just under the surface?’^175^ Despite being ‘sympathetic’, Eric Harper notes that if they had a limb amputated, colonial patients were less likely to fight for their lives. ‘If a Moslem has lost a limb, he will not go to Heaven so he will be frightened of dying’.^176^ According to him, they were also more at risk of being injured, as they were unable to read the warning notices around the minefield.^177^ ‘A little while ago I was interested to observe the tirailleur who works on reception for me, busily engaged in reading a French newspaper – upside down. I did not disillusion him, for perhaps that would have hurt his dignity. He has lent me a book on the Cameroons.’^178^

Despite facing the same risks and hardship as the white members of the hospital, colonial orderlies were demobilised and replaced by French white staff in September 1944, as they were deemed incapable of handling the cold winter of France.^179^ Behind this official justification, based on racial prejudices and theories of martial races, the *blanchiement* (whitening) of the hospital was part of a broader process of removal of soldiers from Sub-Equatorial Africa. This process was in part driven by military concerns about the morale of these troops, the need to integrate internal resisters in the French Army and the necessity to recruit soldiers for Indochina. It was also tied to the necessity to restore France’s imperial prestige and racialised anxieties over the impact of intimate relations between colonial recruits and French women.^180^ Authorities were anxious about the proximity of people of colour to white female ambulance drivers, nurses and French women. In the summer of 1944, French racialised anxieties over French African troops’ sexuality were exacerbated by the scandalous robberies, plunders and rapes allegedly committed by *Moroccan Goumiers* in Italy.^181^

Despite strict discrimination policies and racialised anxieties, emotional bonds were formed between the different communities of the hospital. The sense of shared sacrifice and appropriation of military heroism, even by conscientious objectors and female nurses and drivers, helped develop the legend of the Hadfield Spears. The imagined community of the Hadfield Spears was not fixed and entwined Free French collective imaginary with British cultural ideals (about “character” and the ability to display unflinching good humour in awful circumstances). Belonging to this imagined collective – a franco-colonial-British family – was a small comfort for the staff who faced recurrent military attacks and increased criticisms, as the relations between Edward Spears (Mary Spears” husband) and de Gaulle deteriorated. In June 1945, Vernier reflected on the ways in which Franco-British diplomatic tensions impacted on the day-to-day life of the hospital: ‘We have endured, without any recrimination, the most evident marks of official contempt for the last two years’.^182^ In that way, strategies of resilience were collective, driven by camaraderie and an unspoken pact between the staff. Fear of death, however, occasionally led to the breakdown of this collective structure and emotional distancing.

## A Fragile International Community? Grief, Strategies of Resilience and Relationships

In November 1942, in the forward theatre near El-Alamein, the Egyptian cook Michel Rahmé was killed during an air attack. The French chief of colonial staff was forced to return to the base hospital as his nerves could ‘not take the strain which [was] naturally considerable’.^183^ The medical officer Lepoivre from the Foreign Legion was also seriously injured by a ‘éclat à la poitrine’, but survived thanks to an immediate operation.^184^ During this battle, Jean Vialard Goudou admitted that he was afraid, even though this admission is twinned with a disavowal. ‘I was forcing myself not to show it too much, but I was very afraid’, particularly for my comrades.^185^ El Alamein was a bittersweet victory for the Free French, who lost their chief Amilakvari. Before his death, Vialard Goudou asked him how he could be so fearless ‘Personally, I admit that I was terrified when there were explosions everywhere’. He evokes the profound emotional impact of the loss of their chief. ‘This one, we thought he was invulnerable. We all felt like crying’.^186^ Memoirs of medical staff insist on the necessity to attain psychological and emotional detachment in order to be able to perform medical acts under fire. Obliquely, however, we find some evidence of the difficult emotions that were felt when operating under fire, despite medical training that stressed self-abnegation and emotional control.

During the First World War, Mary Spears coined the term ‘the second battlefield’ to describe the fight of medical personnel to contain and mitigate the suffering of wounded men.^187^ She presented nursing not as a form of healing but rather as ‘part of the military machine, complicit in the mutilation and death of men’.^188^ According to Carol Acton, she articulated her wartime trauma using the ‘language of the diseases and injuries’ she treated.^189^ By contrast, her World War Two Memoir *Journey Down a Blind Alley* is silent about her emotional wounds. Although multiple attacks and challenging environments tested her ‘resilience’ and that of her staff, she stressed how well she and other members of the hospital ‘coped’ with working under fire. She barely talks about the crude decision of medical triage, the guilt of failure to save life or the abandonment of patients. Her emphasis on emotional self-control and abnegation was not atypical: in his oral testimony, Michael Rowntree noted that there were surprisingly few ‘psychiatric problems in the hospital’, with people ‘bearing the situation remarkably well’.^190^ Spears memoirs’ title speaks, however, to the deep sense of disappointment and betrayal towards the Free French that she (and many other British recruits) felt. Instead of showing gratitude, Gaullist elites despised her and regarded her “hospital” as a problematic tool of British propaganda.

As the title *Journey Down a Blind Alley* suggests, an increasingly fraught diplomatic context shaped the Ambulance’s community, making the endeavour of maintaining a sense of togetherness precarious. Initially a symbol of Free-French and British medical cooperation, the Ambulance became embroiled in Franco-British imperial rivalries after 1941. On the ground, British and Free French medical staff faced official contempt. In this context, members of the hospital were eager to defend the name of Spears and show their commitment to their fellow comrades of the First French Division. Tensions between de Gaulle and Edward Spears fuelled resentment towards Gaullist elites on the one hand, camaraderie, and an unspoken pact between medical staff and combatants on the other.

If British and Free French staff’s motives to join the Hadfield Spears’ hospital might have been profoundly different, they nevertheless shared a sense of purpose and worth to the collective effort: most of them (except for colonial orderlies) were not conscripts but volunteers. Further, they were supervised by competent surgeons. The FAU volunteers were extremely close to Colonel Vernier, who took over the direction in May 1942. Vernier’s brother was a pacifist, and he was ‘well acquainted with the outlook of the religious pacifist, though not one himself’.^191^ Vernier saw the hospital community through many difficult moments. In his study of the factors that helped soldiers cope in the Western desert, Jonathan Fennell evokes belief in a cause, leadership, training, quality of weapons and primary group (esprit de corps).^192^ As we have seen leadership was important in the hospital. The Hadfield Spears hospital was the ‘elite’ organization of the Free French, the best supplied hospital at least until 1943, serving under commanders who were eager to save as many lives as possible. Equally important was the culture of emotional restraint prevailing in Allied medicine and described earlier.

Historians can examine the cultural scripts of resilience and the ways in which responses to attacks were culturally prescribed. Important questions remain about the subjective internal mechanisms that sustained individual strength and adaptability when faced with danger and death. As historians have noted, how human beings create and maintain resilience at the individual level poses a ‘mystery’.^193^As Alexander McFarlane reminds us, there is ‘no greater challenge in psychiatry than to predict why some people seemingly flourish and cope in the aftermath of adversity, such as war, whereas other develop severe and long-lasting psychiatric disorders’.^194^ A further difficulty is that of differentiating between the effects of military attacks from other emotional wounds, such as personal bereavement. Many members of the hospital were affected by the death of a loved one, boredom, anxieties about what was happening back home. Mary Spears had lost her daughter to a suicide a few years before the war. The nurse Josephine Pearce lost her fiancé, while the ambulance driver Joselyn Russell’s husband, an RAF officer, died in service. The military records of Pol Thibaux, the heroic surgeon who undertook 21 operations while being bombed in the Western desert, obliquely reveals his inability to cope with his emotional wounds in the aftermath of the war.^195^ It also reflects the violence of the army—as an institution—which could not itself cope with a distressed surgeon. Likewise, after the particularly violent Italian campaign of 1944, the hospital’s log reported a ‘crise de folie’ (Madness crisis) of a colonial sergeant chef who committed suicide after assaulting the adjudant chef Pejout and sergeant Cacherat. Nothing more is said about the circumstances of this incident or the reasons of what might have led to this suicide.

This question of how medical workers as individuals coped has occupied considerable attention in the extensive literature on war, wounding and death, with historians highlighting the importance of a range of factors, from food, writing (letters, diaries…), reading, religious faith, arts and escape mechanisms. Although British military regulations forbade the writing of diaries, keeping a diary was an important way to foster resilience for many members of the hospital: it offered moments of reflection and conversation with the self in a life ‘lived entirely amongst others’.^196^ FAU volunteers and some sisters found comfort in their religious faith, prayers and meetings (for the Friends).^197^ Others were able to build strong friendships and bonds which enabled them to cope with hardship. When André Durbach died of an accident in July 1943, for instance, officers of the division paid tribute to the devotion, ‘smiling friendship’ (amitié souriante), and ‘typically Free French spirit’ of their comrade.^198^ Other accounts hint at the importance of reading and writing letters ‘home’. In the Western Desert, Nev Coates wrote in his diary about reading mail in the lamplight ‘I was taken right home by Dad, Mam and Mary who described Christmas celebrations’.^199^ Letter-writing to family members helped sustaining emotional resilience and the identities that men and women had left behind.^200^ Correspondence could also provide a comforting space to explore new selves and desires.^201^ At times, individuals develop strong emotional attachments with the material and animal environment that surrounded them. The ambulance drivers were extremely attached to their car and their individuality. Rachel Millet recalled that Kit had ‘this dreadful old thing, which was a Chevrolet, which the French christened La Belle Marguerite, which was an absolute sod to drive’ but which Kit adored.^202^ Listening to and playing music also provided comfort for some members of the hospital.^203^The FAU volunteer David Rowlands reminisced about enjoying himself singing carols on Christmas in 1941 broadcasted on BBC radio Levant.^204^ For Michael Rowntree, birdwatching provided solace. He was particularly happy about being able to watch two bird migration in the desert.^205^

The Hadfield Spears formed a community, sustained through social activities and convivial gatherings. Many members of the unit made the most of their leave to do some sight-seeing. In Egypt, the ambulance driver Rachel Millet ‘stayed in great comfort in the luxurious Winter Palace Hotel on the banks of the Nile and forgot all about the war’, riding, playing tennis and crossing the Nile by boat.^206^ Singing, dancing and partying eased the boredom of life at the hospital and offered distraction after periods of intense stress. The pharmacist—Pierre Mergier—was the ‘official barman’ of Spears, who mastered the art of cocktails and the disguise of alcohol behind sugary drinks.^207^ In his memoirs, he notes that after each evacuation of patients, the Hadfield Spears organised a party.^208^ Josephine Pearce recalled the ‘extraordinary’ Christmas party of 1942, when they had ‘far too much to drink’.^209^ For Mergier, of all the parties, the most moving one was in Tunisia in September 1943 when Germaine Sablon sang the *Chant des Partisans*.^210^ Eric Harper’s diary is full of the parties of the hospital, but one seemed particularly memorable. On 3 June 1944, the hospital celebrated the administrative officer’s birthday. ‘Proceedings were opened in fine style by the Colonel who, amidst thunderous applause, heaved his stocky frame up the 12 foot central tent pole. He was followed by the lanky Pere Boillot and by TW, then by a greatly embarrassed Duprey, being more or less pushed up by his feet from the base of the pole by all and sundry’.^211^

FAU volunteers often mentioned the role of humour as agent of psychological resilience and group bonding. During an air attack in Bir Hakeim, Nev Coates wrote that ‘laughter is a good way of relieving pent-up feelings’.^212^ In his oral interview, Roy Ridgway re-enacted Freddy Temple’s voice, revealing how the group deployed comedic understatement to cope with the stress of air attacks near Monte Cassino: ‘I remember hearing this English voice in the darkness of a moment of silence ‘ “ I can’t help feeling that Jerry is going to reply to our little volleys” ’.^213^ This was not limited to the FAU. Although humour is greatly dependent on contextual cognisance and the recognition of common cultural references, members of the hospital found ways to ‘cross’ cultural boundaries and build shared scripts. The role of humour and ‘in-jokes’ within the Hadfield Spears deserves more research, particularly to establish the manner in which it was used as a therapeutic strategy to cross class and gender boundaries and comfort patients.^214^ Humour certainly helped to shape the ‘imagined community’ of the hospital and allow for a limited release from tension and societal inhibition (‘a safety valve’).^215^ It was also a way, as many historians have shown, for patients to negotiate power relations and resist medical power and military discipline within the ‘culture of silence’.^216^ Inside jokes can indeed tell us a great deal about a ‘shared sense of emotional intimacy’ that connected members of a medical community.^217^ This humour was injected in the franglais songs invented by the HS staff and revived after the war.^218^

It is difficult to establish whether and if so, to what extent, sexuality was at play when navigating suffering and developing strategies of psychological resilience.^219^ Romantic relationships did develop between women and men from different social and cultural backgrounds within the hospital. For instance, the nurse Evelyn and the FAU volunteer Jim Cottrell got married in Saintes in the winter of 1944. Evelyn admitted, though, that she never understood Jimmy’s pacifist stance. Prior to marrying him, she broke off the engagement briefly due to her mother’s disapproval of his pacifism.^220^ Rachel Millet also met her husband, a French officer, while working in the hospital. While we can uncover the deployment of romance as a mechanism for maintaining morale, it is much harder to examine the issue of consensual or coerced sexual relations outside the marital frame. In Cairo, the FAU Nev Coates reflected on the ‘vices’ of this ‘Eastern city’: ‘Tony took us to the brothel quarter. […] We talked at length about the evening and pitied the chaps who had nothing else to entertain them whilst in Cairo. I wonder how many of them have girls at home.... Cairo.... seemed to have all the vices of an Eastern city, plus the vices of a city behind a big battle.’^221^ He also mentioned that a French colonial orderlies set up ‘a little business in a dark corner’ of one of the tents of the hospital. ‘He installed an Arab woman and charged his comrades for her services’.^222^

For the Free French, sexual matters could tarnish the high ‘ideals’ for which resisters where fighting. Resisters insisted on the moral grounds of the resistance—precisely at a moment when in occupied France too many French women were suspected of giving themselves to the German victors.^223^ Yet, the high rate of venereal diseases–particularly during the Italian campaign–seems to suggest that sexual encounters took place.^224^ The FAU volunteer Eric Harper notes in his diary that in Vals les Bains a dance was given in honour of the hospital. ‘There were many girls whose menfolk were away, either taken by the Germans or members of the Maquis. There was the task of consoling them’.^225^ Finally, same-sex intimacy left even less archival traces, rendering very difficult to establish whether and to what extent it did occur.

Emotional linkages between members of the hospital were strained during the liberation of France, as the hospital underwent many transformations. French doctors’ heady fantasies of returning to the French homeland proved an anti-climax. On 22 August 1944, the doctor Coupigny made a powerful speech on board the SS Fort Dauphin, which transported the Hadfield Spears from Italy to France. He evoked the emotions of the ‘minute which we all have been waiting for so long’ and the memories of all the comrades who had fallen. Yet, many French members of the Hadfield Spears were disappointed by their encounters with the country they had left four years before.^226^ The forward unit was heavily bombarded (by American planes) on the Canadel beach where it landed. Further, in September 1944, the forced demobilisation of African orderlies—with whom the community had shared the African and Italian campaigns—profoundly transformed the make-up of the community.^227^ In December 1944, the hospital tents were left behind, the hospital losing its autonomy and what made its strength in Africa and Italy. In the bitter words of Mary Spears, ‘the confusion and discord in the hospital reflected what was happening to France’, a ‘medley of discordant elements with her FFI, FTP, her heroic resistance and her bogus resistance, her Pétainists and her milice and her armies from overseas’.^228^

Celebrated as heroes, members of the Hadfield Spears also occasionally faced the bitterness and resentment of the families they had left behind. In 1944, the pharmacist Pierre Mergier took the personal effects of the pharmacist-Captain Rabaté, who had died in 1941 during a sanitary re-supply mission on the road between Damascus and Jerusalem, to his widow. Nev Coates recounted that ‘Madame Rabaté would have nothing to do with Pierre or her husband’s effects. In her opinion he had run away from his responsibilities when he joined the Free French Forces and he [had] ceased to exist for her’.^229^ More importantly, the Hadfield Spears staff witnessed the violent campaign of the Authion and the “sacrificing” of the First French Division just a few days before the end of the war. This particularly bloody and violent battle took place on the Mediterranean coast, where holiday-makers were completely oblivious to it. Members of the Hadfield Spears resented not participating in the last real battle, on the North of the Rhine.^230^ Camped in the luxurious Hotel Bristol in Beaulieu-sur-Mer, in the ‘cruel splendor of sunshine and blue sea’, the hospital suffered its last attack on 24 April 1945: a German boat, full of ammunition, exploded destroying all of the windows of the hospital.^231^ This explosion triggered the interest of the local press, reinforcing the popularity of the hospital and leading to many official visits.^232^ This did not, however, change the international public face of the hospital. On 18 June 1945, Hadfield staff swept down the Champs Elysées in style during the Victory Parade. But Mary Spears wrote bitterly in her diary that her husband had become ‘the enemy of the man he saved’. ‘De Gaulle would have been helpless without him – would not have known what to do – or how to do it.’^233^ Tellingly, when the hospital was abruptly dismantled in June 1945, Mary Spears told the British press that de Gaulle had been angered by the British flags displayed on the cars parading during the victory parade in Paris.^234^Colonel Vernier wrote to General Garbay, commander of the First French Division about that the bitterness felt amongst the staff, who contributed ‘with all their soul from the very early days to de Gaulle’s cause’.^235^ He noted: ‘if in the mind of de Gaulle’s ministers, having served for three years the Ambulance Hadfield Spears constituted an indelible flaw, we will find comfort in the friendship of our *true* friends from the First French Division’.^236^ A year later, Mary Spears’ rancour fuelled the pages of her memoirs *Journey Down a Blind Alley*.

At the Liberation, there was no official commemoration of the Ambulance. The wartime experiences of Hadfield Spears staff could not be easily accommodated within “official” British and French memories of the war. In France, the story of the Hadfield-Spears did not fit in the dominant Gaullist narrative of the Free French: not only was the *Directrice’s* hospital the wife of Edward Spears, regarded as a traitor by the Free French, but her name was a reminder of Free French diplomatic failures in the Levant. Further, the emphasis was on male combatant heroism. The personal archives of Lady Spears contain many letters of gratitude from French officials, but the French state showed little interest in commemorating this multi-national unit, composed partly of British women and Conscientious Objectors. In Britain, Conscientious Objectors also fitted uneasily in the cultural memory of the People’s War, their experiences going against the British popular conception of a just and good war.^237^ British nurses and ambulance drivers generated less interest in popular culture than the female agents of the *Special Operation Executive.*^238^

This did not mean, however, that the strong bonds created during the war disappeared. In stark contrast to the lack of public commemorations, at a personal level at least, some members of the hospital continued to visit each other during their holidays. Former chief medical officer Vernier, despite his busy professional life, spent great energy to go and visit former Hadfield Spears friends. He organised the first post-war reunion in 1964 in Paris.^239^ In January 1969, sixteen Free French came to England for Mary Spears’ funeral. After her death, Free French and British members continued to see each other regularly, adding lines to the Ambulance songs at each reunion. The post-war reunions took place across France and Britain: in Auron (1973), Birmingham (1976), Bendor Island (1979), York (1982), Caudebec-en-Caux (1985), Oxford (1988), Quimper (1991), Newcastle (1994), in Alsace (1996), near Hohwald where the Ambulance was deployed in January–February 1945 and finally, in Frome (1998). At each of these reunions, Mike Rowntree, Neville Coates and David Rowlands sang the Spears’ *chanson de geste* (tune). Jokes and fond memories of the Spears ambulance also regularly appeared in the gazette of the Free French health service (*Bulletin* of the *Amicale de Service de Santé de la France Libre*) in the 1990s.^240^ But, like all forms of post-war reunions, it had its limits: colonial orderlies were not part of this post-war community and only those who could afford it joined this reunion. Some members of the Hadfield Spears felt misrepresented by hegemonic group memories, which romanticised franco-British friendship and the Ambulance’s war experiences. In 1989, for instance, the BBC 2 produced *Tin Hats and Silk Stockings* a documentary on the Hadfield Spears showcasing the experiences of British ambulance drivers, who transformed ‘their life-long passion for horses on to their cars’.^241^ It featured some Free French, nurses and FAU volunteers, who regularly attended the post-war reunions. This documentary angered former British nurses who felt misrepresented and considered that the drivers rewrote themselves at the center of the story, overplaying their own contribution and presenting the war as a fun adventure.^242^ This reminds us that the transnational ethos of stoicism described in this article shaped the ways in which some Spears staff narrated their wartime experiences: it stressed toughness, fortitude and the ability to remain in control at all times, making it difficult to openly voice distress, grief and disagreements.

## Conclusion

This article has unveiled how *Spears* staff negotiated the duality of the spatial environment of the Hadfield Spears as both a space of danger and a home, a site of extreme violence but also deeply compassionate encounters. It has argued that a transnational ‘ethos of stoicism’ emerged in the hospital as it followed Free French troops in the Middle East, Africa and Europe. This ethos manifested itself in a myriad of ways and shaped the ways in which medical workers later remembered and narrated their wartime experiences. In the words of the hospital’s *Directrice,* whether installed in a broken building in Tobruk or tents at Bir Hakeim, Sidi Bouali, or the Lake Bolsena, the Hadfield Spears remained a ‘crossroads between life and death, a place of suffering and laughter that defied it, of torn bodies and trusting eyes, of death defeated by unwavering vigilance’.^243^ The increasingly fraught diplomatic context, combined with the experiences of fire near the front line in Deraa, the Western Desert, Italy and France, shaped and reshaped the hospital’s imagined community, which stressed engagement with the Free French, *British* stoicism and humour, “franglais” sociability and medical bravery. Familiarity with danger and social expectations of bravery did not, however, diminish the distress of dealing with the aftermath of the attacks.^244^Through silences, friendships, and the use of humour, members of the hospital developed individual and collective strategies to confront danger and cope with emotional wounds. In some cases, however, such as that of the ‘hero-surgeon’ of Bir Hakeim Pol Thibaux, it proved hard to shake away the emotional wounds left by the difficulties of caring for dying friends.

A focus on these close ties and emotional bonds does not mean ignoring national specificities nor the tensions between professional groups. It reminds us, as the authors of *The Intimate Life of Dissent* put it, that medical resisters and conscientious objectors, and to a lesser extent nurses and drivers, were not lone individuals solely moved by abstract ideals, but also individuals ‘caught up in other, sometimes contradictory aspirations and relationships and forms of responsibility’.^245^ In addition to adding new historical focus on neglected attacks on healthcare in wartime, this article proposes a fresh scope for combining macro and micro perspectives.^246^ Ultimately, the history of the Hadfield Spears challenges conventional histories of Allied war medicine and the French resistance as intrinsically separate. It reveals obscured connections between Allied official military services and voluntary organizations, between Franco-British imperial rivalries and the experiences of frontline medical evacuation, between the intimate history of pacifism and the ‘emotional’ history of the resistance. By illuminating these neglected historical connections, this article finds that the much lauded ‘ethos of stoicism’ in this medical unit not only bore the imprint of spatially anchored professional values and a fraught diplomatic context but was also fundamentally shaped by the intimate relations between carers from radically different backgrounds. As attacks against healthcare in armed conflict remain an intractable political and humanitarian issue today, this analysis of the social workings of the Hadfield Spears underscores the importance of exploring how intimate encounters under fire shape international diplomatic, military and medical cooperation in time of crisis.

## Footnotes

1 Churchill Archives Centre [CAC], Spears 11/2/5, Brosset à médecin chef de l’Ambulance Hadfield Spears, 18 October 1944.

2 Jacques Duprey, *L’ambulance Hadfield-Spears ou la drôle d’équipe* (Paris: Nouvelles Editions Latines, 1953), 238–39.

3 Cynthia Enloe argues that the armed forces only accept women as long as they remain ‘creatures marginal to the military’s core identity’. *Does Khaki Become You? The Militarisation of Women’s Lives* (London: Pluto Press, 1983), 6.

4 Clotilde de Fouchécour, ‘Un Anglais et le continent’, *Commentaire,* 168, 2019, 837–44.

5 CAC, Spears 11/3/1, Mary Spears’ diary, 18 June 1945.

6 Aviel Roshwald, *Estranged Bedfellows. Britain and France in the Middle East During the Second World War* (Oxford: Oxford University Press, 1990).

7 In 1942, she comments on the difficulties she is facing with Free French medical officers ‘Behind it all is deep feeling against everything British’ CAC, Spears 11/3/2, Spears’ diary, February 1942.

8 Service Historique de la Défense [SHD], 12P231, Général de Brigade Brosset à Monsieur Général de Division, 3 janvier 1944.

9 CAC, Spears 11/2/5, Note de service, Creation et organization de l’ambulance Hadfield Spears, 22 February 1941.

10 The “French side” of the hospital was directed by André Durbach (March–May 1941), Henri Fruchaud (May 1941–May 1942) and Jean Vernier (May 1942–June 1945). Prior to the war, André Durbach was a civilian surgeon, trained in Parisian hospitals and in charge of a surgical team in the Hospital de Janval in Dieppe. Henri Fruchaud was a Professor of Surgery at the school of Medicine of Angers. Jean Vernier was a surgeon (médecin chef) in the Hospital of Bangui (French Equatorial Africa), trained in the school of colonial medicine in Bordeaux (Ecole de santé navale de Bordeaux).

11 Friends’ Library [FL], MSS876, Hist 13, History by Michael Rowntree, 3. For a history of the FAU during the Second World War, see Tegla Davies, *Friends Ambulance Unit: The Story of the FAU in the Second World War 1939–1946* (London: George Allen and Unwin, 1947); for a history of the FAU during the First World War, Fiona Reid, *Medicine in First World War Europe: Soldiers, Medics, Pacifists* (London: Bloomsbury, 2017).

12 Duprey, *L’ambulance Hadfield-Spears,* 67–68.

13 Steven O’Connor, ‘The Free French and British Forces in the Desert War, 1942: The Learning Curve in Interallied Military Cooperation’, *European Review of History: Revue Europeenne d’Histoire,* 27, 1 (2020), 176–98. For 1943–44, Julie Le Gac, *Vaincre sans gloire*. *Le Corps Expeditionnaire Français en Italie (Novembre 1942–Juillet 1944)* (Paris: Les Belles Lettres, 2013), for 1944, Claire Miot *La Première armée française – De la Provence à l’Allemagne, 1944–1945* (Paris: Perrin, 2021).

14 CAC, Spears 11/2/3, Journal des Marches et opérations de l’HCM3, première quinzaine de mai 1945.

15 There is an extremely rich historiography on different applications of primary group theory. Simon Wessely, ‘Twentieth-Century Theories on Combat Motivation and Breakdown’, *Journal of Contemporary History*, 2006, 41, 269–86, Ville Kivimäki and Tuomas Tepora, ‘War of Hearts: Love and Collective Attachment As Integrating Factors in Finland During WW2’, *Journal of Social History*, 2009, 43, , 285–305; Frances Houghton, *The Veterans’ Tale. British Military Memoirs of the Second World War* (Cambridge: Cambridge University Press, 2018).

16 Musée de l’Ordre de la Liberation [MOL], Charles Mauric, unpublished memoir ‘Citoyen du Monde’ [undated], 26.

17 Ibid.

18 Nancy Sherman explored the self-image of the military person as stoical. Nancy Sherman, *Stoic Warriors: The Ancient Philosophy Behind the Military Mind* (Oxford: Oxford University Press, 2005). On medical resilience, Carol Acton and Jane Potter *Working in a World of Hurt*: *Trauma and Resilience in the narratives of medical personnel in warzones* (Manchester: Manchester University Press, 2015); Leo Van Bergen and Eric Vermetten, ed, *The First World War and Health: Rethinking Resilience* (Leiden: Brill, 2020); On methodological reflection on how emotional patterns and practices can vary across the same space, see Benno Gammerl ‘Emotional styles – concepts and challenges’, *Rethinking History, The Journal of Theory and Practice*, 2012, 16,161–75; Amy Bell “Landscapes of Fear: Wartime London, 1939–1945”, *Journal of British Studies,* 2009, 48153–75.

19 On discourses of medical heroism, Margaret H. Darrow, “Making Sister Julie: The Origin of First World War French Nursing Heroines in Franco-Prussian War Stories”, in Alison Fell and Christine Hallett, eds, *First World War Nursing*: *New Perspectives* (London: Routledge, 2013) 17–34; Bertrand Taithe, “Mourir pour des idées humanitaires: Sacrifice, témoignage et travail humanitaire, 1870–990”, in Caroline Cazanave (dir), *Mourir pour des Idées* (Besancon: Presses Universitaire de Franche Comté, 2008), 239–54: ‘Humanitarian Masculinity: Desire, Character and Heroic, 1876–2018’, in Esther Moller, Johannes Paulmann and Katharina Stornig, ed, *Gender Global Humanitarianism in the Twentieth Century* (London: Palgrave, 2021), 35–60.

20 Harini Amarasuriya, Tobias Kelly, Sidharthan Maunaguru, Galina Oustinova-Stjepanovic and Jonathan Spencer, eds, *The Intimate Life of Dissent. Anthropological Perspectives* (London: UCL Press, 2020), 5.

21 Barbara Rosenwein defines emotional communities as ‘social communities […] but the researcher looking at them seeks above all to undercover systems of feeling: what these communities (and the individual within them) define and assess as valuable or harmful to them; the evaluations that they make about others’ emotions; the nature of the affective bonds between people they recognize; and the modes of emotional expression that they expect, encourage, tolerate and deplore’. Barbara Rosenwein, ‘Worrying about Emotions in History’, *The American Historical Review,* 2002, 107, p. 842 and *Emotional Communities in the Early Middle Ages* (Ithaca, NY: Cornell University Press, 2007). Also see Monique Scheer, ‘Are Emotions a kind of practice (and is that what makes them have a history)? A Bourdieuian approach to understanding emotion’, *History and Theory*, 2012, 51, , 193–22; Joachim C. Häberlen and Russel A. Spinney, ‘Introduction’, *Contemporary European History,* 2014, 23, 489–503; Dolores Martin-Moruno and Beatriz Pichel, *Emotional Bodies. The historical performativity of emotions* (Urbana: University of Illinois Press, 2020).

22 Guillaume Piketty, ‘From the Capitoline Hill to the Tarpeian Rock? Free French coming out of war’, *European Review of History: Revue Européenne d’Histoire*, 2018, 25, 358; Tobias Kelly, *Battles of Conscience. The Lives of British Pacifists in the Second World War* (London: Penguin,2022).

23 It is beyond the scope of this article to examine in detail the high principles and different commitments that Hadfield Spears staff made. It is worth, however, pointing that Tobias Kelly has examined the ways in which ‘conscience’ gave meaning and significance gave meaning to the wartime experiences of the FAU group. Kelly ‘Dissenting conscience: the intimate politics of objection in Second World War Britain’ in Amarasuriya, Kelly, Maunaguru, Oustinova-Stjepanovic and Spencer, eds, *The Intimate Life of Dissent*, 112–131.

24 Acton and Potter *Working in a World of Hurt,* 241.

25 On the history of the medical service of the Free French, see SHD, 4P1 Historique des FFL, Commandant Etchegoyen, Chapitre XVIII – La direction du service de santé, Vialard-Goudou ‘Le fonctionnement pratique du Service de santé de la 1ère Brigade de la 1ère DFL pour Bir-Hakeim (27 mai-11 juin 1942)’, *Bulletin de la Société de médecine militaire française,* 1946, 228–34; Jean Vernier, ‘Les campagnes des Forces Françaises Libres’, *Le service de santé dans les combats de la Libération* (Paris: Imprimerie Nationale,1953); Regis Forissier, ‘Les operations militaires des Forces Francaises Libres et leur soutien sanitaire entre 1941 et 1943’, 1993, Val de Grace, Bibliothèque Centrale du service de santé des Armées [unpublished]; Guy Chauliac, *Le Service de Santé de la France libre du 18 juin 1940 au 1*^*er*^*aout* 1943 (Paris: Chauliac, 1994).

26 Mark Harrison, *Medicine and Victory: British Military Medicine in the Second World War* (Oxford: Oxford University Press, 2005).

27 Jean Vialard Goudou, *Journal de guerre d’un médecin de la France Libre* (Paris: Editions du félin, 2022), 136.

28 Forissier, ‘Les operations militaires des Forces Francaises Libres’, 121.

29 I build here on the rich historiography on gender, emotions and war medicine, including Michael Brown “Like a Devoted Army”: Medicine, Heroic Masculinity, and the Military Paradigm in Victorian Britain”, *Journal of British Studies* 2010, 49, 592–622; Ana Carden Coyne, *The Politics of Wounds. Military Patients and Medical Power in the First World War* (Oxford: Oxford University Press, 2015); Jane Brooks, *Negotiating Nursing: British Army Sisters and Soldiers in the Second World War* (Manchester: Manchester University Press, 2018); Jessica Meyer, *An Equal Burden. The Men of the Royal Army Medical Corps in the First World War* (Oxford: Oxford University Press, 2019); Michael Brown, “Wounds and Wonder: Emotion, Imagination and War in the Cultures of Romantic Surgery”, *Journal for Eighteenth-Century Studies* 2020, 43, 239–259.

30 Guillaume Piketty, *Résister. Les archives intimes des combattants de l”ombre* (Paris, Éditions Textuel, 2011); ‘The madonnas of exile: reflections on the emotional life of French external resisters’, plenary conference, University of Manchester, 14 June 2019.

31 Guillaume Piketty ,‘Conclusion in Sylvain Cornil-Frérot, Charlotte Faucher, Laure Humbert, Guillaume Piketty and Thomas Vaisset, ed, *Françaises et français libres. Une identité née de la pluralité* (Rennes: Presses Universitaires de Rennes, forthcoming); Géraud Létang, “Mirages d”une rebellion. Etre Francais libre au Tchad (1940–43)”, These, Institut d’études politiques de Paris, 2019.

32 Henri Fruchaud, Jean Vernier, Paul Guénon, Pol Thibaux.

33 This is not specific to the Free French. Enloe *Does Khaki become you?* 100–101.

34 Mary Spears is aware that De Larminat has doubted about her. Spears 11/3/2, Mary Spears’ diary, 20 February 1942.

35 This was a reference to Madame Catroux, Mary Spears’ rival. Vialard Goudou *Journal de guerre d’un médecin de la France Libre,* 122.

36 , Sonya Rose, *Which People’s War? National Identity and Citizenship in Wartime Britain, 1939–1945* (Oxford: Oxford University Press, 2003), 196; Corinna Peniston-Bird, ‘Classifying the Body in the Second World War: British Men In and Out of Uniform’, *Body and Society*, 2003, 9, 31–48; Linsey Robb and Juliette Pattinson “Becoming Visible: Gendering the Study of Men at War”, in Juliette Pattinson and Linsey Robb and Juliette Pattinson, eds, *Men, Masculinities and Male Culture in the Second World War* (London: Palgrave MacMillan, 2018), 1–22.

37 Kelly, *Battles of Conscience.* Also see Linsey Robb, “The “Conchie Corps”: Conflict, Compromise and Conscientious Objection in the British Army, 1940–1945”, *Twentieth Century British History,* 2018, 29, 411–434.

38 Hugenot, *Le Service de Santé dans les combats de la Libération* (Paris: Imprimerie Nationale, 1953), 171.

39 On nurses, see Brooks *Negotiating Nursing*, 46. There is no study of MTC drivers during the Second World War, but in many respects there were similar to the First Aid Nursing Yeomanry. Janet Lee, “I Wish My Mother Could See Me Now”: The First Aid Nursing Yeomanry (FANY) and Negotiation of Gender and Class Relations, 1907–1918”, *NWSA Journal*, 2007, 19, 148–158; Juliette Pattinson *Women of War: Gender, modernity and the First Aid Nursing Yeomanry* (Manchester: Manchester University Press, 2020).

40 In total, I have examined 11 interviews (8 with men, 3 with women), 3 published memoirs (2 female; 1 male) and 5 private diaries/collection of letters (3 male; 1 female; 1 couple) produced by members of the Hadfield-Spears. In addition, I have analysed diaries of doctors who were not members of the HS, but who were stationed in the same area and worked in close proximity with this medical unit.

41 The archives of this hospital are held in Limoges at the Service des Archives médicales Hospitalières des Armées.

42 Christopher Dillon, ‘Commentary: Masculinity and the Racial State’, *Central European History*, 2018, 51, 513–522, 519.

43 Lyn Smith, *Pacifists in Action: the Experience of the Friends Ambulance Unit in the Second World War* (London: William Sessions Limited, 1998).

44 Penny Summerfield, “Culture and Composure: creating narratives of the gendered self in oral history interviews”, *Cultural and Social History,* 2004, 1, 65–93.

45 For a review of how historians have used these sources, see Penny Summerfield *Histories of the Self: personal narratives and historical practice* (London: Routledge, 2018).

46 Guillaume Piketty, *Francais en Resistance. Carnets de guerre, correspondances, journaux personnels* (Paris: Robert Laffont, 2009).

47 There is a rich literature on the ‘silences’ of resisters, though not specifically examining the meanings of these ‘silences’ for Free French doctors and nurses. See, for instance, Pierre Laborie ‘Silences de la mémoire, mémoires du silence’, in P. Laborie, ed, *Les Français des années troubles de la guerre d’Espagne à la Liberat*ion *(Paris: Desclee de Brouwer, 2001), 53*–71; Laurent Douzou, ‘Les silences d’un résistant’, *Le genre humain* 2012, 53, 21–27.

48 See for instance the conclusion of Guillaume Piketty *Résister. Les archives intimes des combattants de l’ombre* (Paris: Editions Textuel, 2011).

49 Potter and Acton *Working in a world of hurt,* 81–82. Carden-Coyne has also written about how pain broke through the code of silence in First World War Hospitals. *The Politics of Wounds*, 17.

50 Vernier ‘Les campagnes des Forces Francaises libres’, 19.

51 Lucy Noakes, “Communities of Feeling: Fear, Death, and Grief in the Writing of British Servicemen in the Second World War” in Claire Langhamer, Lucy Noakes and Claudia Siebrecht, eds, *Total War: An Emotional History* (Oxford: Oxford University Press, 2020), 116–136, 129; Summerfield *Histories of the Self*; Houghton *The Veterans’ Tale*.

52 Matt Houlbrook, “A Pin to See the Peepshow: Culture, Fiction and Selfhood in Edith Thompson”s Letters, 1921–1922”, *Past and Present,* 2010, 1, 215–249, 226.

53 I would like to thank Katherine Reed for transcribing the three diaries written by Mary Spears (Spears 11/3/1; 11/3/2; 11/3/3). These diaries offer a particularly fascinating window into her sense of helplessness (In May 1941, she wrote ‘I am so bored of it all’) and her anxiety about leaving her husband (In December 1941‘He will have Miss M. instead to look after him + run his house. All wrong. But how can I abandon my hospital job when it goes to the Western desert. […] Stay at home to try and keep B’s affection?’).

54 Margaret Higonnet, *Nurses at the Front: Writing the wounds of the Great War* (Boston, Mass: Northeastern University Press, 2001), xxxiii.

55 CAC, Spears 11/3/1, 18 June 1945.

56 Carden-Coyne *Politics of wounds,* 198.

57 E. Gentili, “Femme et médecin à Bir Hakeim: Louise-Marie Lemanissier”, *Historie des Sciences médicales,* LI, 2017, 3, 371–375.

58 See for instance her broadcast at the BBC during “Demi-Heure Française du Matin”, 20 March 1941.

59 Eric Jennings and Jean-Francois Muracciole have highlighted the central role that Africans played in the French Resistance, while emphasising how difficult it is to grasp the complexity of their identities and perspectives. Eric Jennings, *La France libre fut africaine* (Paris, Perrin, 2014); Muracciole, *Les Français libres*.

60 Marisa J. Fuentes, *Dispossessed Lives: Enslaved Women, Violence and the Archive* (Philadelphia: University of Pennsylvania, 2016).

61 The principles of neutrality and impartiality were not codified until 1965. These principles were ill-defined and contested in the first part of the twentieth century.

62 The bombings of hospitals and hospital ships happened on both sides and was not new. André Durand *Histoire du Comité International de la Croix-Rouge. De Sarajevo à Hiroshima* (Geneva: Institut Henry Dunant, 1978), 8.

63 Service Historique de la Défense [SHD], 12P228, Journal de Marche de l’Ambulance Hadfield Spears. On some of the methodological problems of using official military logbooks as infallible sources of evidence, see Houghton *The Veterans’ Tale,* 59–60.

64 IWM, 7864, Nev Coates *From the London Blitz to the Champs Elysees. An account of Service with the Friends Ambulance Unit, 1940–1945*, unpublished memoir, 17 February 1942, 136. Also see Mary Spears’ diary, 20 March 1942 (Spears 11/3/2).

65 Friends’ Library, FAU 1947/3/5, Notes on the work and conduct of FAU members in Uniform with the Armed Forces.

66 Davies *The Friends Ambulance Unit*, 119.

67 IWM, 18959, Private Papers of E.R Harper, Extracts from War Diary, 5 April 1942, 19.

68 CAC, Spears, 11/2/3, Mary Spears à de Gaulle, 8 juillet 1940; Fruchaud à de Gaulle, 22 octobre 1940, Douala.

69 Henri Fruchaud, “Treatment of Recent War Wounds. French and Spanish Methods”, *The Lancet*, 1942.

70 This is also clear in the testimony of Vialard-Goudou on the health service during the battle of Bir Hakeim ‘Le fonctionnement pratique du service de sante de la 1ere Brigade de la 1ere DFL pour Bir-Hacheim’, *Bulletin de la Societe de Medecine Militaire Francaise*, 1946, 229–234.

71 Vernier, ‘Les campagnes de la France libre’, 16. Chauliac *Le Service de Santé de la France Libre,* 141.

72 Vialard Goudou, *Journal de guerre d’un médecin de la France Libre,* 96.

73 Vialard Goudou, *Journal de guerre d’un médecin de la France Libre,* p. 127.

74 Penny Summerfield, “My Dress for an Army Uniform: Gender Instabilities in the Two World Wars”, Inaugural Lecture, Lancaster, University of Lancaster, 1997.

75 SHD, 12P228, Journal de Marche.

76 Sebastien Albertelli, *Elles ont suivi de Gaulle*. *Histoire du Corps des Volontaires Françaises* (Paris: Perrin, 2020) 74.

77 Claire Miot, “Combattantes sans combattre? Le cas des ambulancières dans la Première armée Française (1944–45)”, *Revue Historique des Armées,* 2013, pp. 25–35; Charles Antoine Wanecq, “Entre le transporteur et l’infirmière: conflits de genre autour de la définition d’un care ambulancier (1939–73)”, *Clio*, 2019, 49, 115–135.

78 Enloe, *Does Khaki become you,* 110.

79 Houghton, *The Veterans’ tale,* 80.

80 Brooks, *Negotiating Nursing*, 97.

81 Vialard Goudou, ‘Le fonctionnement pratique du service de sante’, 231.

82 Francois Broche, *La cathédrale des sables. Bir Hakeim* (Paris: Belin, 2019), 108–09.

83 Nancy Wright Smith, “With the Free French”, *The Canadian Nurse*, 1944, 40, 23–25, 24.

84 On 15 May 1942, the order was received that the Hadfield Spears personnel to be replaced by personnel from the Ambulance Chirurgicale Legere (ACL). For a history of the health service at Bir Hakeim, Vialard-Goudou, ‘Le fonctionnement pratique du Service de santé de la 1ère Brigade de la 1ère DFL pour Bir-Hakeim (27 mai-11 juin 1942)’, *Bulletin de la Société de médecine militaire française*, 1946, 228–234.

85 MOL, Dossier Paul Guénon, Journal, 9 Juin 1942.

86 Vialard Goudou, ‘Le fonctionnement pratique du service de sante’, 232.

87 Chauliac, *Le Service de Santé de la France Libre,* 141.

88 Vialard Goudou, ‘Le fonctionnement pratique du service de sante’, 232.

89 MOL, Paul Guénon, Journal, 1942.

90 MOL, Jean Vialard Goudou, Lettre à sa mère, 129.

91 Jean-Marc Largeaud, ‘Témoignages de Bir Hakeim’, *Revue de la France Libre,* 2012, 44, 2–6.

92 Susan Grayzel, *At Home and under Fire: Air Raids and Culture in Britain from the Great War to the Blitz* (Cambridge: Cambridge University Press, 2012), 252.

93 Bridget E. Keown, “I think I was more pleased to see her than any one “cos she”s so fine”: nurses” friendships, trauma, and resiliency during the First World War”, *Family and Community History*, 2018, 21, 151–165.

94 Duprey, *La drole d’équipe,* 256.

95 In her memoir, Rachel Millet notes that she joined the MTC to follow in the footsteps of her aunt VAD in the First World War *Spearette. A memoir of the Hadfield Spears Ambulance Unit* (Haddenham: Fern House, 1998), 1. Alison Fell *Women as Veterans in Britain and France after the First World War* (Cambridge: Cambridge University Press, 2018).

96 Jean Pictet, “Le droit international et l”activité du Comité international de la Croix-Rouge en temps de guerre”, *Revue internationale de la Croix Rouge et Bulletin international des sociétés de la Croix-Rouge*, 1943, 290, 124–157. On the history of protection, see Larissa Fast *Aid in Danger. The Perils and Promise of Humanitarianism* (Philadelphia: University of Pennsylvania Press, 2014), 69–70.

97 Rainer Baudendistel, *Between Bombs and Good Intentions*: *the International Committee of the Red Cross and the Italo-Ethiopian War, 1935–1936* (London: Berghahn, 2006), 115.

98 Duprey, *La drole d’equipe,* 254.

99 For a study of First World War Ambulance drivers, see Laura Doan, *Disturbing Practices: History, Sexuality and Women’s Experience of Modern War* (Chicago: Chicago University Press, 2013), 120.

100 MOL, Jean Vialard-Goudou, *Journal. Chapitre VII bis*, 110–11. This stereotype of the highly ‘moral’ British upper-class woman also appears in Duprey’s history of the Ambulance. Duprey, *L’ambulance Hadfield Spears*, 255–256.

101 Doan, *Disturbing Practices,* 129.

102 The French planted artillery and camouflaged guns in the middle of hospital, in violation of article 8 of the Geneva convention of 1929, which for the FAU volunteers was “very naughty”. IWM, 10350, Roy Heber Ridgway interviewed by Lyn Smith, 14 July 1987, reel 4, 07”59; also see IWM, 18959, Private Papers of E.R Harper, Extracts from War Diary, 23 May 1944, 55.

103 Mergier and Mergier *Itinéraire d’un Français libre,*vol. 2, 59–60.

104 IWM, 18959, Private Papers of E.R Harper, Extracts from War Diary, 28 May 1944, 56.

105 IWM, 13631, David Rowlands interviewed by Lyn Smith, 15 November 1993, real 4, 06”41.

106 IWM, 10350, Roy Heber Ridgway interviewed by Lyn Smith, 14 July 1987, reel 4, 05”55.

107 IWM, 13631, Rowlands by Smith, 15 November 1993, real 4, 06”41.

108 SHD, 12P231, Le Journal de Marche, 1^er^ avril au 30 juin 1944, signé Vernier.

109 IWM, 10350, Roy Heber Ridgway interviewed by Lyn Smith, 14 July 1987, reel 4, 14”38.

110 SHD, 12P231, Le Journal de Marche, 1^er^ avril au 30 juin 1944, signé Vernier.

111 For instance, the Belgian nurse Simone Buterne, who worked in the Hadfield Spears from 8 October 1941 to 4 August 1942, received several medals (Croix de Guerre avec étoile d”argent; Médaille Coloniale en date du 26.3.42 with mention Erythrée Libye; Médaille de la Résistance and Médaille des FFL) and a legion d”honneur (grade de chevalier). During the battle of Bir Hakeim, she was responsible for plastering the broken limbs of severely injured men without the supervision of a surgeon and under constant bombing. SHD, GR16P98654, Extrait du décret en date du 9 December 1957 portant nomination dans l”ordre national de la légion d”honneur, signé Chaban Delmas; Etat signalétique et des services.

112 CAC, SPEARS 11/2/3, Rapport sur l’activité de l”équipe chirurgicale de l’Ambulance Spears (15 aout 1944-3 septembre 1944), Vernier, 9 September 1944.

113 Duprey, *La drole d’equipe,* 256.

114 Mary Borden, *Journey Down a Blind Alley* (London: Hutchinson & Co, 1946), 169.

115 Amicale des anciens de la Première Division française libre *Annuaire de la Première division française libre et ses unités dans la guerre 1939–45* (Paris: Imprimerie N. Fortin et ses fils, 1972), A-93. This number included members of the ACL, AHS—Ambulance Hadfield Spears, GSD1—Groupe Sanitaire Divisionnaire N.1, 1^er^ BM—1^er^ Bataillon Médical and AFS—American Field service.

116 Duprey, *La drole d’équipe,* p. 267. Two members of the hospital died in an accident in 1941; two from air attacks in 1942 (on 14 February 1942, Raymond Anderson, the British leader of the FAU volunteers is killed by a bombed dropped by the Germans in El Azrag, near Bir Hakeim. on 2 November 1942, the Lebanese cook Michel Rahmé is killed during an air attack near El Alamein). One member of the hospital died drowned (in June 1943, Trochon died on the coast between Zuara and Tripoli, drowned, bathing late at night). In the summer of 1944, the sergent Balla died after a “madness crisis” (crise de folie) and in October 1944, Pejout died in a car crash.

117 The death rate in the FFL is 6.7%. Murraciole *Les Francais Libres,* 268.

118 Davies, *Friends Ambulance Unit,* 125.

119 Acton and Potter, *Working in a world of Hurt,* 87.

120 IWM, 7864, Coates *From the London Blitz to the Champs Elysees,* 133.

121 Agnes Arnold-Foster, ‘Operating with feeling: Emotions in contemporary surgery’, *The Bulletin,* 2018, 100, 12–15.

122 Taithe, ‘Compassion fatigue’, 246.

123 Higonnet, *Nurses at the Front,* xvii.

124 Ana Carden-Coyne, ‘Painful bodies and brutal women: remedial massage, gender relations and cultural agency in military hospitals, 1914–18’, *Journal of War and Culture Studies,* 2008, 1, 139–58.

125 Christine Hallett, *Containing Trauma: Nursing work in the First World War* (Manchester: Manchester University Press, 2009). For Second World War, also see Jane Brooks ‘Nurses as therapeutic agents in the extreme environment of the desert war, 1940–44.’ J*ournal of Advanced Nursing*, 2015, 71, 2520–2528.

126 Sarah Chaney, ‘Before compassion: sympathy, tact and the history of the ideal nurse’, *Medical Humanities*, 2021, 47, 475–484, 480.

127 Ibid.

128 IWM, 831, Josephine Pearce Interviewed by Margaret Brooks, 1976, reel 10, 10”00.

129 Ibid, 12”00.

130 Carden-Coyne, *Politics of Wounds,* 18.

131 Thomas Schich, ed, *The Palgrave Handbook of the History of Surgery* (London: Palgrave Macmillan. 2018); James Kennaway, ‘Military Surgery as National Romance: the Memory of British Heroic Fortitude at Waterloo’, *War and Society,* 2020, 77–92; Michael Brown, ‘Wounds and Wonder: Emotion, Imagination and War in the Cultures of Romantic Surgery’, *Journal for Eighteen Century Studies*, 2020, 43, 239–259.

132 Joanna Bourke, *Fear: a Cultural History* (London: Virago, 2006), 220. For a description of how expectations of courage changed during the Second World War, see Kelly *Battle of Conscience.*

133 For instance, as the hospital received its first baptism of fire in Deraa, Fruchaud remained externally calm. In the words of the pharmacist Pierre Mergier, ‘the intense noise of the nearby explosions left him absolutely stoic [In French *impassible*] and nobody dared to protest’. Pierre Mergier “La campagne du Levant de l’Ambulance Spears”, in Chauliac *Le Service de Santé de la France libre,* 115.

134 Borden, *Journey Down a blind Alley*, 164.

135 Borden, *Journey Down a blind Alley*, 213.

136 See, for instance, Antony Beevor, *D-Day. The Battle for Normandy* (London: Penguin Books, 2009), 259–60 or the heroic account of the famous (if controversial) surgeon Ferdinand Sauerbruch in Berlin in the area of the Berlin Charité. Ferdinand Sauerbruch (transl. by Fernand G. Renier and Anne Cliff) *A Surgeon’s Life* (London: Andre Deutsch, 1953).

137 Jessica Meyer, ‘Neutral caregivers or military support? The British Red Cross, the Friends Ambulance Unit, and the Problems of Voluntary Medical in Wartime’ *War and Society,* 2015, 34, 105-120.

138 Kelly, *Battle of Conscience.*

139 Acton and Potter, *Working in a World of Hurt,* 80.

140 Ben Shephard, *A War of Nerves: Soldiers and Psychiatrists in the Twentieth Century* (Cambridge, MA: Harvard University Press, 2003), 181.

141 MOL, Dossier Henri Fruchaud 377, Proposition pour l’attribution de la Croix de la Libération établie par Catroux, transmise à l’armiral Ortoli le 4 Septembre 1945. Also see Joseph Kessel *Tous n’etaient pas des anges* (Paris: Plon, 1963), 207.

142 Henri Fruchaud, *Chirurgie de guerre* (Beyrouth: Les Lettres Francaises, 1943), vi.

143 IWM, 18959, Private Papers of E.R Harper, Extracts from War Diary, 27 March 1942, 19.

144 He quoted William Butler Yeats’ poem about the Easter rebellion in 1916. IWM, 10350, Roy Heber Ridgway interviewed by Lyn Smith, 14 July 1987, reel 4, 19”33.

145 MOL, Paul Guénon, Diary, 23 mars 1942,1.

146 Ibid, 3.

147 Christian Benoit, Gilles Beötsch, Antoine Champeaux, Éric Deroo (Dir.), *Le sacrifice du soldat. Corps martyrisé, corps mythifié* (Paris, CNRS Éditions, 2009).

148 Douzou, ‘Heros et heroisme’ *Dictionnaire historique de la Résistance,* 939–41.

149 CAC, Spears 11/3/2 Manuscript Diary by Mary Spears Mar [1941]-Mar [1942], February 1942.

150 IWM, 18959, Private Papers of E.R Harper, 18 February 1942, 15.

151 IWM, 7864, Coates *From the London Blitz to the Champs Elysees,* 14 February 1942,134.

152 FAU 1947/3/5, Letter from Mary Spears to Mrs Alderson, 15 February 1942. In 1952, she published a novel where main character is a Quaker *Martin Merriedew*. For her biographer Jane Conway, this novel was a tribute to Nik. Conway *A woman of Two Wars,* 299.

153 Drew Faust, *The Republic of Suffering: Death and the American Civil War* (New York: Alfred A. Knopf, 2008), 30.

154 IWM, 7864, Coates *From the London Blitz to the Champs Elysees,* 14 February 1943, 193.

155 IWM, 13631, David Rowlands interviewed by Lyn Smith, 15 November 1993, real 2, 15”57.

156 CAC, Spears 11/3/2, Mary Borden, diary, 11 June 1941.

157 IWM, 13631, David Rowlands interviewed by Lyn Smith, 15 November 1993, real 2, 17”20.

158 André Francois Lemanissier, typed manuscript (1987), kindly shared by Denis Lemanissier, 20.

159 MOL, Jean Vialard Goudou, Journal de guerre, écrit à Al Faya, January 1942, 22.

160 Borden, *Journey Down a blind Alley*, 156.

161 Janet Watson, “Wars in the Wards: The Social Construction of Medical Work in First World War Britain”, *Journal of British Studies,* 2012, 41, 484–510.

162 IWM, 831, Josephine Pearce interviewed by Margaret Brooks, 1976, reel 1, 12”40. This was already the case during the First World War. Pattinson, *Women of war,* 168.

163 IWM, 12180, Evelyn Cottrell interviewed by Lyn Smith, 09 July 1991, reel 2, 15”00.

164 Pattinson, *Women of war,* 174.

165 IWM, 7864, Coates *From the London Blitz to the Champs Elysees,* 169.

166 CAC, Spears 11/2/4, Confidentiel, Note de Service, Forces Francaises Libres, Hopital Hadfield Spears, 22 July 1941.

167 IWM, 7864, Coates *From the London Blitz to the Champs Elysees,* 89.

168 IWM, 10883, Michael Rowntree interviewed by Lyn Smith, 07 August 1989, reel 4, 12”02.

169 Davies, *Friends Ambulance Unit*, 118.

170 Ibid.

171 IWM, 10883, Rowntree interviewed by Smith, 07 August 1989, reel 4, 12”02.

172 IWM, 7864, Coates *From the London Blitz to the Champs Elysees,* 138–139.

173 Julie Le Gac, “Haunted by Jinns: Dealing with War Neuroses among Muslim Soldiers during the Second World War”, in Xavier Bougarel, Raphaëlle Branche and Cloé Drieu, eds, *Combatants of Muslim Origin in European Armies in the Twentieth Century* (London: Bloomsbury, 2017), 183–203, 188.

174 CAC, Spears 11/2/4, Note de Service, Tirailleurs employés a l”hopital, 2 September 1941.

175 IWM, 7864, Coates *From the London Blitz to the Champs Elysees,* 170.

176 IWM, 18959, Private Papers of E.R Harper, Extracts from War Diary, 17 October 1941, 6.

177 Ibid 20.

178 Ibid, 30.

179 SHD, 12P228, Journal de Marche, 16 September 1944.

180 There is a vast literature on the reasons for the “blanchiement” of the First French Army, see, for instance, Joel Myron Echenberg *Les Tirailleurs sénégalais en Afrique occidentale française, 1857–1960* (Paris: Karthala, 2009); Gilles Aubagnac “Le retrait des troupes noires de la Première Armée à l”automne 1944”, *Revue historique des armées* (1993), 34–46; Fargettas *Les tirailleurs sénégalais*:*les soldats noirs entre légendes et réalités, 1939–1945* (Paris: Tallandier, 2012); Claire Miot “Le retrait des tirailleurs sénégalais de la Première Armée française en 1944. Hérésie stratégique, bricolage politique ou conservatisme colonial?”, *Vingtième Siècle. Revue d”Histoire* 1, 2015, 125, 77–89.

181 Le Gac, *Vaincre sans gloire*; Tommaso Baris « Le Corps expéditionnaire français en Italie », *Vingtième Siècle. Revue d”histoire*, 2007, 93, 47–61.

182 SHD, GR12P228, Colonel Vernier à Général Garbay, in *Journal de Marche*, June 1945.

183 IWM, 18959, Private Papers of E.R Harper, Extracts from War Diary, 3 November 1942, 31.

184 Duprey, *La drole d”équipe,*.84.

185 MOL, Jean Vialard Goudou, Journal de Marche, 139.

186 Ibid, 138.

187 Christine Hallett has demonstrated that in *The Forbidden zone* the reader is let down into the horror of the war itself, forced to feel for the wounded. Hallett, *Nurse writers of the Great War,* 58. In the sketch ‘Blind’, we see how Borden’s distancing mechanism removes her from herself to the point where she observes herself from the outside. Carol Acton and Jane Potter ‘Sticking it’: Resilience in the Life-Writing of Medical Personnel in the First World War’, 357–358.

188 Acton, ‘Negotiating Injury and Masculinity in First World War Nurses’ Writing’, 275.

189 Acton, ‘Obsessed by the Obscenity of War’, 338.

190 IWM, 10883, Michael Rowntree interviewed by Lyn Smith, 07 August 1989, reel 5, 24”“00.

191 Davies, *the Friends Ambulance Unit*, 120.

192 Jonathan Fennell, “Courage and Cowardice in the North African Campaign: the Eighth Army and Defeat in the summer of 1942”, *War in History*, 2013, 20, 99–122.

193 Winter, “Introduction” *Cambridge History of the First World War*, 3, 2.

194 McFarlane, ‘The vexed construct of medical resilience’, 95.

195 His military record contains information about his psychological struggle, which might have been revived by his experiences in Indochina. SHD, GR2000Z20129, Dossier d’officier Pol Thibaux.

196 Houghton, *the Veteran’s tale,* pp. 57–58; Acton and Potter *Working in a World of Hurt,* 90–91.

197 IWM, 7864, Coates *From the London Blitz to the Champs Elysees,* 34.

198 SHD, GR8YE, 40729, Dossier André Durbach.

199 IWM, 7864, Coates *From the London Blitz to the Champs Elysees,* 133.

200 See, for instance, on the First World War: Martha Hanna *Your Death Would Be Mine: Paul and Marie Pireaud in the Great War* (Cambridge: Harvard University press, 2008); Manon Pignot *Allons enfants de la patrie. Génération Grande guerre* (Paris: Seuil, 2012); Michael Roper *The Secret Battle. Emotional survival in the Great War* (Manchester: Manchester University Press, 2009); on the Second World War, Cornelie Usborne “Love letters from front and home: a private space for intimacy in the Second World War?” in Elizabeth Harvey, Johannes Hürter, Maiken Umbach and Andreas Wirsching *Private Life and Privacy in Nazi Germany* (Cambridge: Cambridge University Press, 2019), 280–303.

201 Alison Twells, ‘Sex, Gender, and Romantic Intimacy in Servicemen’s letters during the Second World War’, *Historical Journal,* 2020, 63, 732–753.

202 BBC documentary, *Tin Hats and Silk Stockings*, (1989), 10”07”37.

203 Jean Vernier, *Revue de la France Libre,* 16 Mars 1949.

204 IWM, 13631, David Rowlands interviewed by Lyn Smith, 15 November 1993, reel 2, 28”00.

205 IWM, 10883, Michael Rowntree interviewed by Lyn Smith, 07 August 1989, reel 4, 21:40.

206 Millet *Spearette*, 22–23.

207 Pierre Mergier and Jean Mergier, *Itinéraire d’un Français Libre*, vol. 2, 83.

208 Ibid, 82.

209 IWM, 831, Josephine Pearce Interviewed by Margaret Brooks, 1976, reel 11, 28”50.

210 Mergier and Mergier, *Itinéraire d’un Français Libre*, vol. 2, 54.

211 IWM, 18959, Private Papers of E.R Harper, Extracts from War Diary, 3 June 1944, 57.

212 IWM, 7864, Coates *From the London Blitz to the Champs Elysees,* 142.

213 IWM, 10350, Roy Heber Ridgway interviewed by Lyn Smith, 14 July 1987, reel 4, 00”00.

214 Brooks, *Negotiating Nursing,* 103–06.

215 Jan Rüger, ‘Laughter and War in Berlin’, *History Workshop Journal*, 2009, 61, 23–43.

216 Carden-Coyne, *Politics of Wounds,* 276–286.

217 Houghton, Taking “the jagged edges off”: British Naval Humour during the Second World War” Forthcoming. Jane Brooks, ‘Nurses as therapeutic agents in the extreme environment of the desert war, 1940–44’ J*ournal of Advanced Nursing* 2015, 71, 2520–2528.

218 Smith, *Pacifist in Action,* 137.

219 Carden-Coyne, *The Politics of Wounds,* 250.

220 IWM, 12180, Evelyn Cottrell interviewed by Lyn Smith, 09 July 1991, reel 2, 29”00.

221 IWM, 7864, Coates *From the London Blitz to the Champs Elysees,* 176.

222 Ibid, 169.

223 Jean-Marie Guillon et François Marcot, ‘Amours et sexualité’, Bruno Leroux, Christine Levisse-Touzé, François Marcot (ed) *Dictionnaire historique de la Résistance* (Paris: Bouquins, 2006), 917–18.

224 Le Gac “Le mal napolitain”.

225 IWM, 18959, Private Papers of E.R Harper, Extracts from War Diary, 9 September 1944, 64.

226 Piketty, ‘From the Capitoline Hill to the Tarpeian Rock?’, 354–373.

227 SHD, 12P228, Journal de Marche, 17 September 1944.

228 Borden, *Journey down a blind alley,* 332.

229 Coates, *From the London Blitz to the Champs Elysees,* 368.

230 Mergier, *Itinéraire d’un Français Libre*, 2, 110.

231 Borden, *Journey Down a blind Alley,* 352.

232 Mergier, *Itinéraire d’un Francais libre,* vol 2, 113.

233 CAC, Spears 11/3/1, Mary Spears Diary, 18 June 1945.

234 ‘De Gaulle Angered by Union Jack’, Lady Spears*, Dundee Courier, 25 June 1945.*

235 SHD, GR12P228, Vernier à Général Garbay, June 1945.

236 Ibid.

237 Robb, “The Conchie Corps”, 411–34.

238 Pattinson, *Behind Enemy Lines,* 1–24.

239
*La mémoire des Français Libres: Hommes et Combats* (Paris: Publication de la Fondation de la France Libre, 2000), 2940.

240 These bulletins are available in the archives of the Fondation de la France Libre (Paris).

241 BBC documentary, *Tin Hats and Silk Stockings*, (1989), 10”08”11.

242 IWM, 12180, Evelyn Cottrell interviewed by Lyn Smith, 09 July 1991, reel 2, 15”30.

243 Borden, *Journey Down a Blind Alley,* 351.

244 IWM, 13631, David Rowlands interviewed by Lyn Smith, 15 November 1993, real 3, 09”30.

245 Amarasuriya, Kelly, SMaunaguru, Oustinova-Stjepanovic and Spencer, eds, *The intimate life of dissent. Anthropological perspective,* 3.

246 For a recent overview of the literature on Attacks on Healthcare, see Rohini J, Haar, Róisín Read, Larissa Fast, Karl Blanchet, Stephanie Rinaldi, Bertrand Taithe, Christina Wille, and Leonard S. Rubenstein ‘Violence against healthcare in conflict: a systematic review of the literature and agenda for future research’, *Conflict and health*, 2021, 15, 1–18.

